# Organon der Heilkunst

**Published:** 1846-01

**Authors:** 


### Art. XXI.

1. *Organon der Heilkunst.* Von Samuel Hahnemann. *Dresden,* 1819. 8vo, pp. 574.

2. *Fragmenta de Viribus Medicamentorum positivis, sive in sano corpore humano observatis.* A Samuele Hahnemann, m.d. Edidit T. F. Quin, m.d.—*Londini,* 1834. 8vo, pp. 214.

3. *Samuelis Hahnemanni Materia Medica Pura, sive Doctrina de medicamentorum viribus in corpore humano sano observatis, e Germanico sermone in Latinum conversa.* Conjunctis studiis ediderunt Dr. Stapf, Dr. G. Gross, et E. G. à Brunnow.—*Dresdæ,* 1826-28. 2 vol. 8vo, pp. 450, 378.

4 *Die Chronischen Krankeiten, ihre eigenthümliche Natur und homöopathische Heilung.* Von Dr. Samuel Hahnemann. *Dresden,* 1828-30. 4 Bände, 8vo, pp. 241, 362, 312, 407.

5. *Pharmacopœia Homœopathica.* Edidit T. F. Quin.—*Londini,* 1834. 8vo, pp. 164.

6. *Homœopathy Unmasked; being an Exposure of its principal Absurdities and Contradictions: with an Estimate of its recorded Cures.* By Alexander Wood, m.d. *Edinburgh,* 1844. 8vo, pp. 196.

7. *An Introduction to the Study of Homœopathy.* Edited by J. J. Drysdale, m.d. and J. R. Russel, m.d.—*London,* 1845. 8vo, pp. 253.

8. *An Inquiry into the Homœopathic Practice of Medicine.* By W. Henderson, m.d., Professor of Medicine and General Pathology in the University of Edinburgh.—*London and Edinburgh,* 1845. 8vo. pp. 238.

9. *Homœopathic Domestic Medicine.* By J. Laurie, m.d. Third Edition. —*London,* 1846. 8vo, pp. 576.

Although the subject of Homœopathy has been but little adverted to, and never formally noticed, in the pages of this Journal, we have not been unaware of its claims to attention, nor regardless of its remarkable progress in every country of Europe, both as a system of medical doctrine and a system of medical practice. We ought probably to have noticed the subject long ago. At any rate, we can refrain no longer from doing so—now, when one of the publications whose title heads this article, shows that the new doctrine has found its way into the halls of one of our most estimable universities, and is openly advocated and promulgated by its professor of pathology. On the present occasion, however, we do not intend to examine the homœopathic doctrine fully or systematically: this we may probably do on another occasion, and at no distant date. All that our other engagements and the space now at our command will permit, is, to lay before our readers some hasty sketches and some fragmentary views relating to the general subject, which have long occupied our thoughts, and which are now, as it were, forced from us somewhat suddenly and prematurely by the perusal of Dr. Henderson's book.

SAMUEL CHRISTIAN FREDERICK HAHNEMANN, the author of Homœopathy, was born at Meissen, in Saxony, in the year 1755, and died at Paris, only three years since, in the eighty-eighth year of his age. No careful observer of his actions or candid reader of his writings, can hesitate for a moment to admit, that he was a very extraordinary man,—one whose name will descend to posterity as the exclusive excogitator and founder of an original system of medicine, as ingenious as many that preceded it, and destined, probably, to be the remote, if not the immediate, cause of more important fundamental changes in the practice of the healing art, than have resulted from any promulgated since the days of Galen himself. Hahnemann was undoubtedly a man of genius and a scholar; a man of indefatigable industry, of undaunted energy. In the history of medicine his name will appear in the same list with those of the greatest systematists and theorists; unsurpassed by few in the originality and ingenuity of his views, superior to most in having substantiated and carried out his doctrines into actual and most extensive practice. Nor will the overthrow of his system, as a system, deprive him of his fame, so long as Paracelsus, and Stahl, and Silvius, and Boerhaave, and Brown, and the other hundred heroes of theoretical renown, are remembered by their successors in the schools of medicine.

The thoroughly radical change in the theories and practice of medicine, propounded in the system of Hahnemann,—a change equivalent to a total reversal and subversion of almost all that had preceded it,—naturally roused great and general opposition to it in the minds of medical men. This, and the seemingly-monstrous extravagance of one of its main dogmas—that of infinitesimal doses—so abhorrent at first sight to common sense, and so obnoxious to the attacks of a facile ridicule—has, up to this day, prevented common justice being done to the new system, and to its author and his successors. By most medical men it was taken for granted that the system was one, not only visionary in itself, but was the result of a mere fanciful hypothesis, disconnected with facts of any kind, and supported by no processes of ratiocination or logical inference; while its author and his apostles and successors were looked upon either as visionaries or quacks, or both. And yet nothing can be further from the truth. Whoever examines the homœopathic doctrines as enounced and expounded in the original writings of Hahnemann, and of many of his followers, must admit, not only that the system is an ingenious one, but that it professes to be based on a most formidable array of facts and experiments, and that these are woven into a complete code of doctrine with singular dexterity and much apparent fairness. And it is but an act of simple justice to admit, that there exist no grounds for doubting that Hahnemann was as sincere in his belief of the truth of his doctrines as any of the medical systematists who preceded him, and that many, at least, among his followers, have been and are sincere, honest, and learned men. That there are charlatans and impostors among the practitioners of Homœopathy cannot be doubted; but, alas, can it be doubted, any more, that there are such, and many such, among the professors of orthodox physic?

On these grounds, then, it appears to us reasonable, that the claims of Homœopathy, regarded as a system of medical doctrine, ought to be admitted so far as to entitle it to investigation, at least; and, in undertaking such an investigation, we have no more right to reject the evidence supplied in its favour by its professors, than we have of rejecting any other evidence in favour of any other medical doctrine, theoretical or practical.

The first idea of the fundamental doctrine of Homœopathy, seems to have entered the mind of Hahnemann in the year 1790, (the forty-fifth year of his age,) while engaged in translating Cullen's Materia Medica into German. Dissatisfied, it is said, with the author's attempt to explain the action of bark in curing intermittent fevers, he resolved to make trials with it on his own person,—he being then in perfect health. Having taken a sufficient quantity of this drug, he affirms that he was speedily attacked with symptoms resembling those of ague; "and forthwith," says his historian, "arose in his mind a conception of the great truth which was destined to constitute the basis of the new art of medicine." * 'May not,' he reasoned, 'the power of cinchona to cure ague, depend on its power to excite in the healthy body a similar disease?' With the view of testing the truth of his hypothesis, he tried the effects of other medicines on himself and others, and always, it is said, with the same result, viz. "that the medicines excited in the healthy body the same symptoms which they were capable of removing when these occurred naturally in the diseased body." Proceeding then to examine the records of medicine, as to the effects accidentally produced by poisons and other strong drugs, and finding everything, as he believed, confirmatory of his own views derived from experiment, he hesitated no longer to consider as established, and to promulgate the grand and universal law, that "every (dynamic) disease is best cured by that medicine which is capable of producing in the healthy body similar symptoms, or a similar disease, (ὅμοιον παθος;")† or, as it is usually stated more briefly, *similia similibus curantur—Like are cured by like*, i.e. *homœopathically*. The doctrine was hence named *Homœopathy*, and those who adopted it *Homœopathists*, or *Homœopaths*. In contradistinction, the common medical doctrine was named, from employing, in the treatment of disease, medicines producing an effect not *like* (ὁμοιος,) but *different* (αλλος) from that produced by the disease, *Allopathy* (αλλο παθος), and its professors Allopathists or Allopaths. It is convenient, for the sake of brevity, to make use occasionally of these terms. Possessed of this, as he conceived, unfailing clue to all the mysteries of therapeutics, he and his disciples commenced an extensive and long-continued series of trials of the effects of various medicines on their own persons, and on the persons of others. The results of these experiments are recorded in Hahnemann's 'Fragmenta de viribus Medicamentorum positivis,' and 'Reine Arznei-mittellehre,' or 'Materia Medica Pura,' —the former first published in 1805, the latter in 1811. The results of the whole of these proceedings was regarded by Hahnemann as confirming, in every case, his great primary law, and as extending its application practically to a vast number of diseases. All that was requisite, hence

* Miro symptomatum utriusque morbi concentu tactus, magnam statim præsagivit veritatem quæ novæ artis medicæ fundamentum facta est. (*S. Hahnemanni Materia Medica Pura.* Dresdæ, 1826. Introductio Edit. p. vi.)

forth, to the successful treatment of diseases, was the selecting the medicine whose effects on the healthy body came nearest the symptoms of the particular disease to be treated. This selection was rendered easy, as to numerous diseases,—i.e. so far as the experiments had gone,—by reference to the published records of the experiments; and the knowledge was to be extended by further trials of the same kind with other medicines.

Hahnemann gave this as the *rationale* of the cures thus effected, viz. that of two similar actions developed in the same part, the stronger destroys the weaker; but he regarded his doctrine as substantially based *on experience*, and therefore as independent of any theoretical explanations. The curing of diseases *homœopathically*, that is, with medicines producing similar symptoms in the healthy, was, he maintained, *a fact* which could not be disputed, whether the theory invented to explain it was true or false.

It would appear that the doctrine of *infinitesimal doses* constituted no original or necessary part of the general doctrine of homœopathy. In complete accordance both with the theory and the primary experiments, medicines might be given homœopathically, and still in appreciable doses. And all the accounts we find in the writings of Hahnemann, on the origin and establishment of this part of his doctrine, strike us as being much less explicit than might have been expected on a point of such essential importance, and which has always constituted so prominent a feature of his system. He merely informs us in his *Organon*, (§ 240 et seq.) that it being found injurious, in the treatment of diseases, to produce a *medicinal malady much* greater than the *natural malady* intended to be cured, the object of the practitioner should be to produce an affection in the least possible degree greater than that to be removed, so that when the latter vanishes, the former may leave no trace behind: in other words, the energy of the medicament being expended in extinguishing its hostile *double*, none is left to harm the constitution of the patient. But owing to the remarkable sensibility of the diseased body to the agent producing a like action in the part affected, it is, he says, very conceivable how an extremely minute dose of a well-chosen remedy should suffice to produce the necessary degree of action. And experience, he assures us, verifies this presumption; it being found, on trial, that it is hardly possible to attenuate too much the dose of a remedy, provided it be well chosen. "It is of little consequence that this attenuation may go so far as to appear impossible to common physicians, whose minds are only conversant with gross material notions. Vain declamations (he truly adds) must cease in the presence of an experience that cannot err." (§ 278.) In the fourth edition of the 'Organon,' he tells us that experience had led him to diminish the doses much more than he thought necessary at first; and this smallness of dose, astounding as it is, now constitutes, as we have already said, one of the most striking parts of the practice of homœopathy, and is, indeed, now universally considered as inseparable from it, and even an essential part of it.

The consideration of this reduction of the homœopathic doses, from a sensible to an infinitesimal amount, suggests to the sceptical or suspicious mind another explanation of the cause much less favorable to

Hahnemann's views. It may be said, for instance, that while medicines were administered in sensible doses, on the homœopathic principle, *similia similibus*, they were found to act *not* beneficially, because any effect they produced was, at best, not curative, and, probably, was injurious by disturbing the curative effects of nature. When they were reduced to infinitesimal doses, they ceased to produce *any* effect on the system, and so came to *seem* beneficial by not interfering with the *vis medicatrix*.

There seems also to be a contradiction in the facts, as well as the reasoning of Hahnemann, in regard to this matter. He says it is from the sensitiveness of the affected part being exalted to an extraordinary pitch by the disease, that the remedy operates in the infinitesimal dose. If this is the case, how does he explain the alleged facts, on which all his therapeutics is based, viz. the production of such a multitude of symptoms (i.e. medicinal diseases) in the healthy body, as recorded in his 'Materia Medica Pura,' and his 'Fragmenta?'

Every one has heard of this incomprehensible posology; but we are inclined to believe that few, if any, but the homœopathists themselves, or those who have read their books, (and only a small number have) are aware of its infinite and astonishing minuteness. What passes respecting it, in common medical parlance, is regarded as a playful exaggeration of the truth, garnished good humouredly for the nonce, like the ornamental facts of the story-teller. And it is no wonder that this is so. Mere imagination, working primarily on its own ground, could never have reached such a climax of the marvellous. Here, assuredly, if anywhere, the truth, if truth it be, is stranger than fiction.

So minute are the doses prescribed by the Hahnemannic school, that they are scarcely conceivable by the human mind. They defy all the powers of chemistry and physics to detect in them any trace of the remedial substances which they profess to contain, and they almost confound arithmetic in reckoning their amount. We are not ashamed to confess, that our own powers are inadequate to put down in figures an ordinary homœopathic dose, and we suspect that many of the homœopathists themselves would find themselves in the same predicament on trial. The following are the different *attenuations* or doses used :

| | | | |
|---|---|---|---|
| First | = | one hundredth of a drop or grain. | |
| Second | = | one ten-thousandth | do. |
| Third | = | one millionth | do. |
| Sixth | = | one billionth | do. |
| Ninth | = | one trillionth | do. |
| Twelfth | = | one quadrillionth | do. |
| Fifteenth | = | one quintillionth | do. |
| Eighteenth | = | one sextillionth | do. |
| Twenty-first | = | one septillionth | do. |
| Twenty-fourth | = | one octillionth | do. |
| Twenty-seventh | = | one nonillionth | do. |
| Thirtieth | = | one decillionth | do. |

The primary dilutions or attenuations are used comparatively rarely; the higher ones, as the sixth, twelfth, twenty-first, and thirtieth, very commonly. It may be worth a moment's trouble to try how far we really understand or comprehend these numbers. Looking at the first of these we have no difficulty. The *hundredth* (100th) part of a grain, is intelligible enough; the *ten-thousandth* (10,000th) is comprehensible, but begins to waver before the mental view; while the *millionth* (1,000,000th) part of a grain, puts our powers of comprehension on the rack, and leaves us in a chaos of undefined entities or non-entities, we know not which. We fancy that we grasp the reality, and then it instantly vanishes as a phantom, even beyond the sphere of imagination itself. Having got so far, the additional subdivisions, or attenuations, scarcely add to our difficulties. The mind, in any such case, is occupied by a word more than a thing,—and whether the word be a Millionth, Billionth, or Decillionth, the power of comprehension seems the same. And yet the *actual difference* between these quantities is immense,—so immense as to be almost as inconceivable as the actual things themselves. This will be more intelligible, we think, by setting it down in words thus :—

One thousand thousands, is . . . A Million.
One million millions* . . . . . . . A Billion.
One million billions . . . . . . . . A Trillion.
One million trillions . . . . . . . . A Quadrillion.
And so on to . . . . . . . . . . . A Decillion.

Now, we believe this last denomination (according to the English mode of numeration) would stand thus in figures :—

1,000000,000000,000000,000000,000000,000000,000000,000000,000000,000000,000000,000000.

Imagine, if you can, a grain of silica, or charcoal, or oyster shell, (powerful remedies, according to Hahnemann and his followers, in this attenuated condition,) divided into this number of parts: and one of these parts is not only a fit and proper dose to be given as a remedy for severe diseases, but is an agent of such potent influence on the animal economy, that *one dose* of this amount will continue acting for thirty, forty, or fifty days, and must not be interfered with by any repetition of it, for fear of deranging or destroying its curative virtue! Thus, Hahnemann tells us that a sextillionth of a grain of carbonate of ammonia will act beneficially upwards of thirty-six days ;† that the decillionth of a grain of oyster shell (*calcarea*) will require forty, fifty, and even more days, "to effect all the good it is capable of ;"‡ that a similar dose of plumbago (*graphites*) will act for at least from thirty-six to forty-eight days :§ and a like dose of phosphorus, at least forty days.‖ "Of such minute division," remarks Dr. Alexander Wood, in his very clever pamphlet, "no language can give even the slightest idea, and though calculations may express it in figures, yet they fail to convey any mental conception of the amount." He accordingly gives the following analogical illustrations, as tending, at least, to help us to comprehend the unbounded vastness, or, rather, infinite littleness, of the subject contemplated, if not to compass themselves in our minds.

"A billion of moments have not elapsed since the [Mosaic] creation of the world, and, to produce a decillion, that number must be multiplied by a million seven separate times. The distance between the earth and the sun is ninety-five millions of miles; twenty of the homœopathic globules, laid side by side, extend to

* This is according to the English mode of calculation. The French calculate by thousands—not by millions; e. g. with them a billion is a thousand millions only.

† Die Chronischen Krankheiten, band ii, p. 20. ‡ Ib. p. 67. § Ib. p. 148. ‖ Ib. iii, p. 48.

about an inch, so that 158,400,000,000 of such globules would reach from the earth to the sun. But when the thirtieth dilution is produced, each grain is divided into 100,000; 000,000; 000,000; 000,000; 000,000; 000,000; 000,000; 000,000; 000,000; 000,000 parts,* so that a single grain of any substance, in the thirtieth dilution, would extend between the earth and the sun 1,262; 626,262; 626,262; 626,262; 626,262; 626,262; 626,262; 626,262; 626,262 separate times!" (p. 108.)

After this, the more familiar illustrations that one hears of, such as a grain or drop of the original medicine being dissolved in the lake of Geneva, the Caspian, or the Mediterranean, and then a drop of the marine solution given as a homœopathic dose, will hardly appear extravagant.

It is, however, but justice to Hahnemann and his followers to state, that they only then attribute such powers to their infinitesimal doses, when the remedies are prepared in a peculiar manner; maintaining that new properties and powers are developed in them by the frictions and shakings to which they are thereby subjected. The evidence they adduce in support of this opinion, is entirely derived from *experience,* they say: medicines prepared in their peculiar manner being found capable of curing diseases, while, if otherwise prepared, they are not.

The character of this evidence may be more particularly considered hereafter; we will only now remark, that its validity will depend entirely upon the quality of the evidence which they can adduce under the name of *experience.* If they adduce no other proof but the fact of diseases ceasing on or after the employment of their medicines, the fact, though repeated *ad infinitum,* if standing simply by itself, must go for nothing in the way of proof. If they can show a sufficiently large number of instances of two parallel series of diseases, the one series treated homœopathically, the other left to nature, and show that all or the vast majority of the one set were cured or benefited, and the other set not,—then, indeed, we shall be prepared to admit the conclusiveness of the argument based on experience. And in this case we must concede to the Homœopathists, that no argument based on the mere ground of a positive inconceivableness of a dose, or a supposed impossibility of its action, will have any weight. "Empty declamations," to repeat Hahnemann's own words, "must give way before the might of infallible experience."

The doctrine of infinitesimal doses, based, as it is, on the alleged infinitesimal sensitiveness of the diseased body, or, at least, of the affected part, must, as a matter of course, draw after it, as a corollary, the necessity of a strict regimen during the cure of diseases. If medicinal substances, reduced below the standard of mental conception, are able to produce such great effects on the animal system, *a fortiori* may many other things entering the body in the shape of food or drink, or acting on it from without, produce similar, or, at least, somewhat analogous effects, to the great detriment of the individual and the utter counteraction or derangement of the remedial process instituted by the homœopathic medicament. To be sure, it might be argued that, as the former class of substances are not "prepared" according to the homœopathic formula, they ought not to act so energetically. But to this it is replied, that many of the substances in question are taken in such large doses, that they affect the system *allopa-*

* We believe Dr. Wood is here under the mark, and that the real sum is that given by us above.

*thically*, or, in other words, openly and palpably. And this is, indeed, true. It was therefore necessary, on the principles of the new doctrine, that this matter of regimen should demand the strictest attention. In *acute* diseases, mania excepted, Hahnemann advises the instinctive desires of the patient to be complied with, in regard to food, drink, temperature, &c.; but in *chronic* diseases a rigid system of exclusion is enforced.

This fact, the peculiar *homœopathic regimen*, is one of great importance in every point of view, and must never be lost sight of in our attempts to estimate the value of homœopathy as a system of therapeutics.*

So far, it must be allowed, the doctrines of Hahnemann have either a show of reason in themselves, or, at least, claim to be founded on grounds even superior to reason—experience and experiment. There is, however, an important part of the system founded by him, which is essentially hypothetical, and which it is hardly possible for even his own disciples, especially those educated in this country, to assent to. This is his doctrine of the origin and nature of *chronic diseases*. Hahnemann maintains that all these, or with hardly an exception, are derived from three cutaneous diseases, *syphilis, sycosis,* and *psora,* or common itch. Of the whole class of chronic diseases, he attributes one eighth part to the two former maladies, and the remaining seven eighths to the last. In nearly all chronic diseases, then, the real object of consideration with the physician, and the thing to be cured, is not the ostensible diseases, but their all-pervading cause and basis, the psora, or itch. This psora he considers to be originally a disease of the whole system, which only shows itself locally on the surface in its progress. If it be cured in this form by local means, it infallibly gets worse internally, and may long subsist in this condition—for many years even—before it puts on the semblance of any formal chronic disease. In its amorphous state it may be so latent, that, although existing for years, the patient is entirely unaware of being out of health. Often, however, it is productive of a vast variety of obscure symptoms, which are seldom attended to, but which are recognizable by the adept. It is curable in this state; but it is seldom submitted to cure until it has declared itself under the guise of some more formal chronic malady. Then the patient seeks relief from medicine; and wo be unto him if he is treated by an allopath, or even by a homœopath on the general principles of homœopathy! No: it is not sufficient that the remedies prescribed are *similia similibus* simply; they must be taken from a special circumscribed class, named *anti-psoric,* discovered by Hahnemann to be gifted with a specific virtue for the cure of the itch and the itch-diseases.† It is laid down as an axiom, that *nature cannot possibly cure any of these*

* The following is a list of things forbidden, given in a note to § 260 of the 'Organon;' coffee, tea, beer, drinks containing aromatics, spiced chocolate, scents and perfumeries of all sorts, tooth-powders (liquid or pulverised) containing aught medicinal, perfumed bags, high-seasoned meats, ices and pastry flavoured with aromatics, all herbs and roots having medicinal properties, cheese, meats too long kept, pork, goose, duck, too young veal. The following things are also prohibited: over-indulgence at table of all kinds, too much salt or sugar, all spirituous drinks, over-heated rooms, sedentary life, passive exercise on horseback or in a carriage, sleep after dinner, sexual pleasures, exciting books, uncleanliness, anger, vexation, scorn, exciting play, over-exertion of mind, marshy districts, confined localities where the air is stagnant, &c.

† In a note appended to the first section of the 'Chronischen Krankheiten; 'On the Nature of Chronic Diseases,' we have an enumeration of some of the diseases derived, from psora. This list contains the names of *all* our common chronic diseases, of which 120 are formally named.

*chronic diseases;* but that, unless treated properly, that is, homœopathically and anti-psorically, they must infallibly get worse until they end in death. And it would seem that, although always curable by the proper remedies, they are as chronic in their cure as in their nature. The anti-psorics may work *tuto et jucunde*, but hardly *cito*, since we find Hahnemann declaring that no one but a quackish ignoramus can fancy that a disease of so long standing can be cured in a few weeks;* or, if so cured, it is only for a time, to burst out with ten-fold fury by and bye. If this doctrine be the truth, we are sorry for the many patients with chronic diseases cured so rapidly by Dr. Henderson. By this time, we fear, he will have them all again on his list, in the full horrors of this universal original sin of psora. As the disease may be of ten or twenty years' standing in the body of the unconscious patient, Hahnemann says that a cure effected in one or two years must be considered as rapid. Some of our allopathic readers will be the less surprised at this *festina-lente* proceeding, when they are informed that the true anti-psoric treatment in such cases forbids the repetition of the single decillionth dose first prescribed, until after the lapse of twenty, thirty, forty, or even fifty days! The total number of anti-psoric remedies detailed in the treatise on Chronic Diseases is twenty-two. Mercury is considered as a false anti-psoric, and its employment denounced as producing the most dangerous consequences; often, indeed, benefiting speedily, but only for a time; the disease returning in a vastly greater degree or worse form.† And here, again, we fear that our friend, Professor Henderson, who employs this medicine in these psoric diseases, must have forgotten the instructions of his master, and must look forward to the relapse of some of his best cases, so triumphantly but unscientifically cured by "*Mercurius* 6."

The preceding is a brief outline of the main doctrines of Homœopathy; very imperfect indeed, and confessedly doing injustice to the large and important subject; yet, it is to be hoped, accurate as far as it goes, and assuredly drawn up honestly and candidly. It is not our intention, on the present occasion, to submit the doctrine to any minute or formal critical examination: before, however, proceeding to the notice of Dr. Henderson's book, we wish to make some cursory remarks upon it in its double aspect, as a system of doctrine, and as a practical art.

As has been already stated, we think it impossible to refuse to homœopathy the praise of being an ingenious system of medical doctrine, tolerably complete in its organization, tolerably comprehensive in its views, and as capable of being defended by feasible arguments, as most of the systems of medicine which preceded it. It is quite another consideration whether it is TRUE.

If homœopathy can defend itself with more feasible arguments than many of its opponents imagine, it is assuredly obnoxious to objections which it cannot easily rebut. These may be found in ample detail in Dr. Wood's pamphlet, and in many other books and journals of easy access to the reader. We would here indicate a few of the more important which must present themselves to most minds in considering the general question.

* "Nur ein gewohnlichen, unwissender Curirer kann leicht versprechen, eine schwere, langwierige Krankheit in 4, 6 wochen zu heilen." (Kron. Krank. B. I. 230.)

† Chronischen Krankheiten, B. ii, p. 12.

1. We hold the great alleged fact from which the doctrine took its rise, to be no fact at all; or, at least, not to be a fact of that generality of manifestation, which a theory said to be of universal applicability, ought to rest upon. We deny, on the one hand, that many of the medicines said by Hahnemann to be capable of exciting artificial diseases, or the symptoms of diseases, in the healthy body, are really possessed of such powers. We instance, in proof of our assertion, the very medicine which gave rise to the idea of the doctrine in its author's mind—cinchona. We deny that it will produce ague, or anything like ague, or any other form of fever, in the majority of human beings; and so of a large proportion of the homœopathic remedies in most common use. On the other hand, we affirm that some medicines are capable of curing morbid conditions of the body which are incapable of exciting any such condition in the healthy body.

2. We affirm that a large proportion of the experiments performed by Hahnemann and his friends, with the object of ascertaining the therapeutic properties of medicines, are altogether fallacious; and that the alleged facts thereby elicited are not facts at all. We believe that of the numerous—we had almost said innumerable—symptoms recorded in their trials, the vast majority bore no other relation to the medicaments swallowed, but the relation of sequence. Not a shadow of *proof* exists that the symptoms were the consequence or direct effect of the medicine; while a thousand reasons can be adduced for supposing the contrary to be the fact. As the doses administered in these trials—at least, in the later and principal trials—were administered in infinitesimal doses, we are fully warranted in even denying entirely that *any* effect was produced by them. Before we can be called on to admit the recorded phenomena as *consequences* of the medicines, we have a right, as in the case of the treatment of diseases, to call for a parallel series of healthy persons set down to record all their sensations for days, after taking *no* medicines. This the homœopathists cannot give us. In these experiments it seems to be taken for granted that every bodily or mental change, every sensation, every action that occurred subsequently to the medicine being taken, was *caused* by the medicine. Every feeling and occurrence was recorded, and everything is admitted as matter of course. Yet no unprejudiced person, who examines these records even superficially, can for a moment believe that one half or one tenth of the symptoms recorded, were, or could be, produced by the medicaments swallowed. The very *number* of the symptoms stated to be produced, independently of their character, suffices to show the absurdity of the conclusions drawn. Thus, for example, 1090 symptoms are recorded as effects of oyster-shells (calcarea); 590 as produced by plumbago; 1242 as the effects of the ink of the cuttle-fish (sepia). If we had room to give specimens of the various symptoms, no doubt could remain on any candid mind as to the utter want of any necessary connexion between a vast proportion of them and their alleged causes. Among these symptoms thus alleged to be produced, we have almost every sensation which man or woman can feel, derangement of nearly every function of the body, and many formal diseases, *surgical* as well as medical.

3. Even in the cases where positive effects are produced on the healthy body by medicines in sensible doses, these effects (except in a very small number of instances) bear a most imperfect resemblance to any natural malady, or even to the *symptoms* of any malady. Several formidable diseases may, indeed, be said to have no symptoms—as, for example, those diseases which are called *latent*. How many diseases have been detected only on dissection after death, and which have escaped the recognition of the most experienced physicians? Every physician, for example, has met with cases of chronic pleurisy with extensive effusion into the chest, which presented *no pectoral* symptoms, and which were only detected by auscultation. How could the fitting remedy for such cases be selected on the principle of *similia similibus?*

4. Many persons deny the truth of the homœopathic therapeutics, on the mere ground of the extreme improbability of the *theory* of disease adopted by the homœopathists. We do not admit the validity of this objection. If we once admit that the homœopathic doses possess a medicinal potency, and that this potency exerts itself in exciting actions analogous to those of certain diseases, we see nothing unfeasible in the doctrine that the new artificial action should destroy the previous natural or morbid one. At least, this is as good and rational theory as most of our orthodox medical theories. And, indeed, it is supported by several strong analogies afforded both by pathology and (allopathic) therapeutics.

5. But to admit the potency of the homœopathic medicaments is not so easy. Indeed, it is so difficult, that all the arguments that have hitherto been adduced in support of the affirmative of the proposition, are incapable of making any impression on ordinary minds, while the glaring improbability of the fact lies open before them. All the arguments of weight seem to be on the other side; and nothing but the demonstration of the truth—if truth it is—by positive physical facts within the sphere of the senses, can ever win assent to it. The reasons against the doctrine are so manifold and obvious that it is almost unnecessary to state them. That substances possessing a power of acting on the animal economy in doses of a certain appreciable amount, and which are found to lose their power when administered in quantities still appreciable but less than this amount, should once more acquire the same or similar properties, when this lesser quantity is rubbed for a few minutes in a mortar, or shaken for a second or two in a phial, would be a thing most strange and unaccountable. That when the quantity was reduced not merely below an appreciable amount, but so far below this as to vanish utterly from the senses and set at defiance all power of detection, and almost of calculation;—nay, that when attenuated to such a degree as to be inconceivable by the human mind, the substance should not only regain the potency it had lost, but a potency vastly greater—would surely be still stranger and still more unaccountable. But when—going far beyond all this—we find the homœopathist maintaining that substances utterly powerless in a state of sensible bulk, even in the greatest amount, acquire astonishing powers by mere subdivision, without any discoverable change in their physical or chemical properties,*—can any proposition be submitted to human apprehension that seems more utterly improbable—more ludicrously absurd? To be called on to believe that the decillionth of a grain of charcoal or oyster-

* It will be afterwards seen that Hahnemann says the chemical properties *are* changed by attenuation; but the arguments he brings in proof are invalid.

shell, is capable of producing hundreds of the most formidable symptoms, and of curing, as by magic, the most inveterate diseases—while we can take ounces, nay, pounds, of the very same substance into our stomachs with no other inconvenience than its mechanical bulk—seems so gratuitous an outrage to human reason that the mind instinctively recoils from the proposition.

It is, however, but fair to give the reader an opportunity of exercising his own judgment on this question, by stating the precise nature of the manipulations to which the remedies are subjected, and under which these marvellous powers are said to be developed. It is also reasonable that he should be made aware of the kind of arguments by which it is attempted to explain the manner in which so extraordinary a change takes place, or rather to illustrate, by analogy, its possibility at least, if not its probability. This we shall now do.

We translate the following directions (which must be rigidly followed,) from the introduction to Hahnemann's work on psoric or chronic diseases.

Ninety-nine grains of sugar of milk are pulverized and divided into three parts, each of course containing 33 grains. If the medicament to be prepared is solid, one grain, if liquid, one drop, is added to one of the parts of sugar of milk in an unglazed porcelain mortar: the two substances are mixed together for a moment, by means of a horn or bone spatula, and are then rubbed (with a middling degree of force—mit einiger Kraft) with the pestle, also unglazed, for *six minutes:* the mass is then scraped [by the bone or horn spatula we presume] from the bottom of the mortar and the pestle, during the space of *four minutes* more: it is then rubbed as before *six minutes: four minutes* are again consumed in scraping the mass together. The second portion of sugar of milk is then added, the two are stirred with the spatula for an instant, and are then subjected for six minutes to similar trituration. The powder being again scraped together during the space of four minutes, is once more triturated for six minutes, and this time more forcibly (kräftig gerieben). Being again scraped for four minutes, the third and last portion of the sugar of milk is added; the whole is mixed by the spatula, and then forcibly triturated for six minutes; again scraped four minutes and again triturated six. The powder is then carefully removed from the mortar and pestle and deposited in a stoppered phial. This is the *First degree of attenuation,* or the hundredth degree of power.

To raise the medicament to the *Second degree of attenuation,* or the 10,000th power, one grain of the powder thus prepared is mixed with one third of ninety-nine (thirty-three) grains of sugar of milk; these being well stirred with the spatula, are forcibly triturated for six minutes and scraped for four; and the same operations are performed on adding the second and third portions of the sugar of milk respectively. The powder is then preserved as before in stoppered phials.

It thus appears that each attenuation is effected by means of six triturations of six minutes each, and six scrapings of four minutes each; the whole period of preparation occupying exactly the space of one hour.

To obtain the *Third attenuation* (the *millionth*) a grain of the second attenuation is taken and treated precisely in the same manner. The higher attenuations are obtained from this third-power powder, by means of solutions in alcohol and water, and are thus effected. In the first place, one hundred drops of strong alcohol and one hundred drops of distilled water, both of low temperature (keller-temperatur, cellar-temperature) are mixed together by means of *ten shakes of the arm* (mit 10 Arm-Schlägen).* One grain of the powder of the third or millionth attenuation being placed in a phial, a hundred drops (or one half) of the diluted alcohol is poured upon it, the stoppered phial is then turned slowly round on its axis (um seine Axe langsam gedrehet) for some minutes, until the powder is dissolved, and then twice shaken.

The next dilution or attenuation is formed, by adding one drop of the preceding to ninety-nine drops of pure alcohol, and giving the phial containing these, two shakes. The next attenuation is formed precisely in the same manner, by adding one drop of the preceding solution to ninety-nine drops of alcohol, and consummating the union by the same double arm-shaking. And all the higher dilutions are obtained exactly in the same way, one drop of the immediate predecessor constituting the hundredth part of its successor.

The shakings must be of moderate force, and in order that they may be uniform, the phials must be of such a size as to be exactly two thirds filled by the liquid.

Finally, in order to fit the medicines for actual administration, fine globules of sugar of milk are prepared, (as near as possible of the same size and about that of a poppy seed) two hundred of which weigh one grain or thereabouts. These globules are all moistened with the proper attenuation, by being touched by the moistened stopper of the phial containing it, and are themselves preserved dry in stoppered phials, ready for being swallowed in such numbers as are prescribed.†

We cannot find in Hahnemann's writings any explanation of or reason for the *precise* and *peculiar* mode and amount of the manipulations prescribed. He, however, gives in many places reasons why, or, at least, analogical illustrations how, it may result, that the rubbings and shakings, added to the infinitesimal subdivision, may confer on the substances operated on, the new properties ascribed to them, the acquisition of which he admits to be almost miraculous, and conferring on homœopathy, and especially on himself, for having made the discovery, no slight honour and glory.‡

In proof of this he instances the solubility of substances in water, spirits of wine, &c., which were previously insoluble in any such medium, such as petroleum, oyster shells, silica, the metals, &c.; the non-alteration of substances previously alterable when exposed to certain agents, as phosphorus exposed to air, neutral salts exposed to acids, &c. In illustration of the power of infinitesimal doses to act on the human body, he instances the matter of contagion, magnetism, animal magnetism, &c.

* This is the direction in the text, but in a note the author says, that, for several years past he had employed only *two shakings* instead of ten (ein zweimaliges Schütteln mit zwei Arm-Schlägen statt des Zehnmaligen) having been convinced by many comparative trials on the sick that the lesser number is not only sufficient but preferable. He adds, though not very intelligibly, that two shakes develop as great an *amount* (Menge) of medicinal power, though in a lower *degree* (nicht in so hohem Grade). (Band II, s. 10.)

† Chronischen Krankheiten. b. ii, pp. 5-11.

‡ Ibid. b. ii, s. 1-2.

One or two obvious remarks suggest themselves in reference to what immediately precedes. The consideration of the *peculiar* manipulations inculcated in the preparation of the medicines, can hardly fail to produce an impression very unfavorable to the author of them. In the first place, it is manifestly *impossible* for any human being, during the course of a long life, much less in the course of a few years, to have performed a sufficient number of experiments, or made a sufficient number of comparative trials, to enable him to state with any degree of certainty, that these particular manipulations and none others, were the exact and exclusive means to produce the effect desired. Thousands and tens of thousands of instances would be insufficient, as could be shown mathematically, to enable the experimenter to decide whether there should not, for example, be *three* shakings instead of *two*, or whether the triturations and the scrapings should not be each of *five* minutes, instead of the one being *six* and the other *four*. In the second place, it certainly has a very suspicious look of a foregone conclusion, rather than of a legitimate deduction from facts, that all the scrapings and rubbings to which each remedy is subjected, in each single stage of its transmigration, should occupy exactly *one hour* and not one minute more or less. (That time as well as the degree of friction, &c., is not a matter of indifference in Hahnemann's estimation, is obvious, from the change he was induced to make in the number of shakings, from the original ten to two.) And then the slow turning of the phial on its axis, the directing one set of triturations to be stronger than another, &c. Altogether, it must be admitted, that the whole complexion of the thing bears a much closer resemblance to what we have heard or seen of magical ceremonies and the tricks of conjurors, demonstrations for effect and to produce an impression, than to any operation of a scientific or *bona fide* character.

As to the argument founded on the alleged solvency of substances previously insoluble, it must go for nought, as it is well known that many substances are found in nature dissolved in minute quantities, or, which is the same thing, diffused in particles of invisible dimensions, in water and other fluids, although they are not soluble in mass or under ordinary circumstances.

Many other objections to the doctrines of homœopathy have been made; and it would not be difficult to add to their number; but enough has been already said to prove its unsoundness as a theory; and if it came before us only as a theory, it would be unnecessary to waste more time in the discussion of its merits. The days are long past in medicine, when anything merely theoretical could claim prolonged attention. No doctrine, however ingenious, not based on positive demonstrable facts, will any more be regarded but as a piece of poetical speculation, which may indeed amuse the fancy, but can never influence the conduct of scientific men, much less of practical physicians. But homœopathy comes before us in a much more imposing aspect, and claims our attention on grounds which cannot be gainsaid. It presents itself as a new art of medicine, as a mode of practice utterly at variance with that long established in the world; and claims the notice of mankind on the irresistible grounds of its superior power of curing diseases and preserving human life. And it comes before us now, not in the garb of a suppliant, unknown and helpless, but as a conqueror, powerful, famous, and triumphant. The disciples of Hahnemann are spread over the whole civilized world. There is not a town of any considerable size in Germany, France, Italy, England, or America, that does not boast of possessing one or more homœopathic physicians, not a few of whom are men of high respectability and learning; many of them in large practice, and patronised especially by persons of high rank. New books on Homœopathy issue in abundance from the press; and journals exclusively devoted to its cause are printed and widely circulated in Europe and America. Numerous hospitals and dispensaries for the treatment of the poor on the new system have been established, many of which publish Reports blazoning its successes, not merely in warm phrases, but in the hard words and harder figures of statistical tables. The very fact of the publication of a *third* edition of such a large and expensive work as Dr. Laurie's (No. 9 of our list,) proves how widely the practice is spread among the public generally. The last triumph which homœopathy has achieved, is the conversion of the Professor of Pathology in the University of Edinburgh from the old faith.

As an established form of practical medicine, then; as a great fact in the history of our art; we must, nolentes volentes, consider homœopathy. If, as is maintained by its advocates, it is indeed true, that with its infinitesimal doses it cures diseases; nay more, that it cures them exactly according to the ancient *beau ideal* formula, *tuto cito et jucunde;* and cures them also in a larger proportion than is done by ordinary treatment; it matters but little whether its theory is false or true. If it can *prove* to us, that it does what we have just stated, we are bound to admit, and we are prepared to admit, that this is a kind of evidence sufficient to overthrow all the arguments we can bring against it, however strong, and all our reasonings, however just: improbabilities, however glaring, and even what seem impossibilities, must go for nothing. As Dr. Henderson truly says,

"It is in vain that physicians attempt to oppose the system by commenting on the flaws in the hypotheses formed to explain it, the incidents which are said by its founder to have led him to the discovery of what is peculiar in it, or the alleged blunders of its practitioners. There is no hypothesis in homœopathy that is of the smallest consequence to the practice of it. The question now is, not whether it originated in a mere speculation, or an induction of facts, but whether it be, as actually employed in the treatment of disease, a valuable acquisition to the practice of medicine; and it is of little consequence to the fundamental importance of the system, that its practitioners should be chargeable with occasional errors of diagnosis, as great, or greater, than those which are every day committed by others." (p. 44.)

In this point of view, then, what has homœopathy to present to us?

The subject here to be considered naturally divides itself into two parts: 1st, As to the *absolute power* of homœopathy to cure diseases: 2dly, As to its *power relatively to that of allopathy.*

1. In regard to the first head of the inquiry, we think we are justified in stating that no unquestionable evidence exists as to the absolute power of homœopathy to cure diseases. The only way in which this power could be effectively established, would be by the institution of an experiment, on the large scale, on two sets of parallel cases of disease, the one treated homœopathically, the other treated *apparently* in the same manner, but with fictitious globules in lieu of the real globules of homœopathy. An experiment of this sort properly conducted on a sufficiently large number of persons, for a sufficiently long period, would settle the question of the absolute potency or impotency of the homœopathic treatment. At present, we have no such experiments conducted on a sufficiently large scale to render the result valid. Such experiments as have been made—and several have been made in the German hospitals—must be considered, as far as they go, as unfavorable to the claims of homœopathy. Experiments made in private practice, in a small number of cases, at most, are still less entitled to consideration : as far as they go, the results obtained by them also tell against homœopathy. Nevertheless, we are disposed to waive this evidence on the positive side of the question, as being inadequate, and therefore conclude, as above, that we have no unequivocal evidence to prove that medicines administered homœopathically, and in homœopathic doses, have a positive power of curing diseases.

2. On the second head of the inquiry, our evidence is very different both in character and amount. Here homœopathy can adduce evidence of precisely the same kind as allopathy. The homœopathic evidence, however, is so much less than the allopathic, in absolute amount, that we must declare, *in limine,* that it is quite insufficient to enable us to come to a sure conclusion on *the whole question* at issue between the parties. Much too short a period has elapsed since the establishment of homœopathy, for it to possess the requisite data that could enable it to contend with an opponent which has at command the accumulated materials supplied by millions of observers during an experience of two thousand years. And even admitting, as we readily do, that a vast part of those materials is utterly valueless, still it would be unfair to put them in competition with the scanty evidence furnished by a few observers during a few years,—which evidence, moreover, is precisely of the same general character as that of the older school, and consequently deteriorated by the same proportion of what is inadmissible. Nevertheless, it would surely be most unwise, and even unphilosophical, to come to the conclusion, that, because we are not yet in a position to decide the question absolutely and definitively, we should therefore refuse to entertain it at all. Matters that immediately and nearly concern human health, human happiness, or human life, cannot be so treated. And therefore it is, that, imperfect as the data are, we feel bound not to dismiss the subject of homœopathy without a brief inquiry, at least, into its pretensions and merits as a branch or form of practical medieine. In doing so, it is quite unnecessary, after what has just been stated, to sift *all* the evidence the new doctrine can supply us on this head; for the purpose we have in view, the materials contained in two of the books before us are quite sufficient.

The Hospital of the Sisters of Charity in Vienna was opened in 1832. It is situated in a healthy suburb, and has thus advantages over the great general hospital of the same city. It contains at present upwards of fifty beds. In the beginning of 1835, the management of the hospital was committed to Dr. Fleischmann, and since that period all the patients have been treated according to the homœopathic system exclusively. In the Introduction to the Study of Homœopathy, by Drs. Drysdale and Russel, there is a translation of a report of Dr. Fleischmann, exhibiting a tabular view of the cases treated at this hospital during eight years—from the beginning of 1835 to the end of 1843. The total number of patients treated was 6551, and the following are the general results:

| | |
|---|---|
| Remaining from 1834 . . . . . . . . . | 27 |
| Admitted . . . . . . . . . . . . . . | 6524 |
| Cured . . . . . . . . . . . . . . . | 5980 |
| Dismissed uncured . . . . . . . . . . | 112 |
| Died . . . . . . . . . . . . . . . | 407 |
| Remaining . . . . . . . . . . . . | 50 |

The list includes all the usual diseases, acute and chronic, found in hospitals, and some surgical cases. The following extract shows the number and events of some of the more important and best marked diseases:

| | Admitted. | Cured. | Uncured. | Died. | Recovered. |
|---|---|---|---|---|---|
| Abscess of the brain . . . . | 3 | . . | . . | 3 | |
| Apoplexy . . . . . . . . | 9 | 4 | 2 | 3 | |
| Cancer of stomach and uterus . | 5 | . . | 2 | 3 | |
| Amenorrhea and Chlorosis . . | 90 | 89 | . . | . . | 1 |
| Ascites . . . . . . . . | 14 | 10 | 1 | 3 | |
| Diarrhœa . . . . . . | 114 | 112 | . . | 2 | |
| Dysentery . . . . . . | 44 | 42 | . . | 2 | |
| Erysipelas of the face . . | 181 | 177 | 1 | 2 | 1 |
| Fever, excluding typhus . . . | 1036 | 1007 | 1 | 17 | 11 |
| Typhus, abdominalis . . . | 819 | 669 | 2 | 140 | 8 |
| Influenza . . . . . . | 52 | 51 | . . | 1 | |
| Dyspeptic affections . . . | 173 | 172 | . . | . . | 1 |
| Gout, acute and chronic . . . | 102 | 97 | 1 | 4 | |
| Headaches, various . . . | 61 | 61 | | | |
| Articular inflammations . . . | 211 | 203 | . . | 2 | 6 |
| Meningitis . . . . . . | 17 | 15 | 1 | 1 | |
| Bronchitis . . . . . . | 15 | 15 | | | |
| Ophthalmia . . . . . . | 51 | 30 | 1 | | |
| Endocarditis . . . . . . | 29 | 29 | | | |
| Pericarditis . . . . . . | 2 | 2 | | | |
| Enteritis . . . . . . | 6 | 1 | . . | 5 | |
| Pneumonia . . . . . | 300 | 280 | . . | 19 | 1 |
| Peritonitis . . . . . . | 105 | 100 | . . | 5 | |
| Pleuritis . . . . . | 224 | 221 | . . | 3 | |
| Measles . . . . . . . | 25 | 23 | . . | 2 | |
| Phthisis . . . . . . . | 98 | . . | 27 | 71 | |
| Rheumatism, acute and chronic . | 188 | 188 | . . | . . | |
| Scarlatina . . . . . . . | 35 | 31 | . . | 2 | 2 |
| Smallpox . . . . . . . | 136 | 120 | . . | 11 | 5 |
| Tonsillitis . . . . . . | 300 | 299 | . . | . . | 1 |

It is well known to all physicians accustomed to statistical inquiries, that, without a minute classification of the individual diseases included in any general report of cases, showing the sex, age, condition of the patients, the precise character or *genius* of the prevailing diseases, the season, the date of the disease when brought under treatment, &c. &c., no trustworthy comparison can be instituted between any two lists of diseases, however similar in name, and although occurring in the same locality. The difficulty of comparison will, of course, be considerably enhanced, when the countries, nature of the localities, general habits, &c. &c. of the patients, are different in the two cases. It would, therefore, lead to no useful purpose to institute any close comparison of Dr. Fleischmann's bare skeleton tables with any similar tables of diseases treated allopathically in this country or elsewhere. The conclusions deducible from such a comparison, whether for or against either mode of treatment, could not be admitted as of any positive weight in settling the practical question at issue. To enable us to do this effectually, we would require from each party an incomparably greater number of cases, observed and treated through a long period of time, and each disease discriminated, and all classified according to the rigid requirements of statistics. We do not, however, mean to say that such lists as those of Dr. Fleischmann's are unworthy of notice and incapable of furnishing any information of consequence. This is not the case. Although yielding us no positive results or such data as science demands, they unquestionably furnish us with isolated facts of great value, and even supply materials which may be worked into such rude approximations to truth, as medicine has, alas, been too long content withal. These tables, for instance, substantiate this momentous fact, that all our ordinary curable diseases are cured, in a fair proportion, under the homœopathic method of treatment. Not merely do we see thus cured all the slighter diseases, whether acute or chronic, which most men of experience know to be readily susceptible of cure under every variety of treatment and under no treatment at all; but even all the severer and more dangerous diseases, which most physicians, of whatever school, have been accustomed to consider as not only needing the interposition of art to assist nature in bringing them to a favorable and speedy termination, but demanding the employment of prompt and strong measures to prevent a fatal issue in a considerable proportion of cases. And such is the nature of the premises, that there can hardly be any mistake as to the justness of the inference. Dr. Fleischmann is a regular, well-educated physician, as capable of forming a true diagnosis as other practitioners, and he is considered by those who know him as a man of honour and respectability, and incapable of attesting a falsehood. We cannot, therefore, refuse to admit the accuracy of his statements as to matters of fact; or, at least, to admit them, with that liberal subtraction from the favorable side of the equation, which is required in the case of all statements made by the disciples and advocates of new doctrines. Even after this rectification, we see that enough remains to justify the inference above deduced. No candid physician, looking at the original report, or at the small part of it which we have extracted, will hesitate to acknowledge that the results there set forth would have been considered by him as satisfactory, if they had occurred in his own practice. The amount of deaths in the fevers and eruptive diseases is certainly below the ordinary proportion; but, for reasons already stated, no conclusion favorable to homœopathy can be thence deduced. It seems, however, reasonable to infer that, even in these cases, the new practice was not less favorable to the cure than the ordinary practice. In all such cases, experienced physicians have been long aware that the results, as to mortality, are nearly the same under all varieties of allopathic treatment. It would not surprise them, therefore, that a treatment like that of homœopathy, which they may regard as perfectly negative, should be fully as successful as their own. But the results presented to us in the severer internal inflammations, are certainly not such as most practical physicians would have expected to be obtained under the exclusive administration of a thousandth, a millionth, or a billionth part of a grain of phosphorus, every two, three, or four hours. It would be very unreasonable to believe that, out of 300 cases of pneumonia, 224 cases of pleurisy, and 105 cases of peritonitis, (in all 629 cases,) spread over a period of eight years, *all* the cases, except the fatal ones, (27 in number,) were slight, and such as would have seemed to us hardly requiring treatment of any kind. In fact, according to all experience, such could not be the case. But, independently of this *a priori* argument, we have sufficient evidence to prove that many of the cases of pneumonia, at least, were severe cases. A few of these cases are reported in detail by Dr. Fleischmann himself, and we have ourselves had the statement corroborated by the private testimony of a physician (not a homœopath) who attended Dr. F.'s wards for three months. This gentleman watched the course of several cases of pneumonia and traced their progress, by the physical signs, through the different stages of congestion, hepatization, and resolution, up to a perfect cure, within a period of time which would have appeared short under the most energetic treatment of allopathy.

In examining Dr. Fleischmann's report, the sagacious physician will not fail to be struck by the fact, that the relative proportion of cures, and the relative mortality of the different diseases, one to another, are precisely the same as he is accustomed to see in his own practice. Slight diseases are all cured by the homœopathist, as by the allopathist; dangerous maladies kill a considerable proportion of the patients of both; very dangerous ones, a still larger proportion; and the class of diseases which all true observers and honest reporters have declared rebellious to their most strenuous medical efforts, are found to occupy the same black column in the tables of the old and the new school.

Thus: the cases of dyspepsia (173), the cases of headache (61), the cases of chlorosis (90), the cases of tonsillitis (300), the cases of simple rheumatism (188), the cases of bronchitis (15), are ALL *cured;* while we have 1 death in 52 cases of influenza, 2 deaths in 114 cases of diarrhea, 2 deaths in 181 cases of erysipelas of the face, 2 deaths in 211 cases of arthritis. As we advance to the still more dangerous class of cases, we find the loss proportionably greater: thus, out of 44 cases of dysentery we have 2 deaths, out of 9 cases of apoplexy we have 3 deaths; out of 14 cases of ascites 3 deaths, and 1 not cured; out of 1036 cases of ordinary fevers we have 17 deaths, while out of 819 cases of typhus we have 140 deaths; out of 524 cases of pneumonia and pleurisy we have 22 deaths; out of 105 cases of peritonitis 5 deaths, out of 136 cases of small-pox 11 deaths, out of 35 cases of scarlatina 2 deaths, and out of 6 cases of enteritis 5 deaths; while *all* the cases of cancer (5), *all* the cases of abscess of the brain (3), and *all* the cases of phthisis (98), are either registered as fatal, or as "dismissed uncured,"—which, of course, means the same thing. The only cases in the list which do not seem, at first sight, to come within the above category, are the cases of endocarditis and pericarditis (31), which are all reported as cured. These are, no doubt, severe diseases; and this may seem an uncommon amount of success; yet, when it is considered that the number of cases is not great, that the diagnosis of endocarditis, and even of pericarditis, is less easy and certain than that of many other diseases, and that it is not so much in their primary condition as in their ultimate effects, that these diseases are dangerous, we believe that even the degree of success here recorded cannot, in fairness, be admitted as any deviation from the ordinary course of events in allopathic medication.

The remarks above made are even of more importance, in relation to the general subject now under consideration, than they may seem to be at first. They not only show that the *kind* of successes and failures experienced by the homœopathists, is precisely the same as that experienced by the allopathists; but they also seem to show that the medication of the former can boast of no *peculiar* virtue whereby it can achieve triumphs in fields altogether forbidden to the latter. Under the influence of medicines, all of which must be considered *new*—new absolutely, or new in their form, mode of administration, and principle of action,—we would have hardly expected to find the old relations of curability and incurability exactly preserved. Does not this fact, common to both, seem to point to a *community of power, or want of power,* in the two classes of agents, rather than to a speciality of action and potency in one?

The materials furnished by Professor Henderson towards the solution of the practical portion of the question agitated between the old and new systems, are very inferior in amount and in intrinsic value, to those of Dr. Fleischmann. They are, however, not unimportant; and they are the more to be prized as the evidence they supply is of a somewhat different kind from that adduced by the German physician.

The first part of Dr. Henderson's book consists of general observations deprecatory of the wholesale condemnation of homœopathy by the medical profession; of apologetical statements in relation to his own secession from the established faith; of arguments in defence of his own views against the accusations of his opponents; and a detail of reasons why homœopathy ought to be studied and tried, at least, if not embraced by the professors of medicine generally. This introduction is written in a philosophical, fair, and candid spirit, and bears the impress of sincerity. It is, however, a production of no great power, and is, moreover, disfigured by a vicious style. It is a tolerably fair specimen of that cloudy and verbose style first introduced by Dr. Chalmers, and which has seduced so many Scottish writers from the path of plain English. In this sort of writing, you gather the author's meaning rather from the impression conveyed by any passage as a whole mass of words, than by the direct communication of definite ideas by words and phrases of precise import. It is to the ordinary language of lettered Englishmen, what the mountains of Scotland, when enveloped in November mist, are to the same mountains when standing out clear and defined in the sun of June: you know the mountain is there, you recognize its broader features, but you see nothing clearly and distinctly.

The second part of Dr. Henderson's book, which alone we have here to do with, consists of a detail of one hundred and twenty-two cases of disease treated homœopathically by Dr. Henderson, in dispensary and private practice. They all bear the impress of being faithfully related, though most of them are deficient in essential details, and many of them are utterly valueless to any class of inquirers. Dr. Henderson seems to have exercised his usual fairness in selecting the cases for publication :—

"I have" he says, "contented myself with adhering strictly to the following determination in regard to the details that I should publish, namely, that they should consist of every case of which an account was written at the time it first presented itself, and, of course, before anything was known of the effects which might succeed treatment. That determination, in respect to every case, so taken down, whether successful or not, has been fulfilled . . . . and I think a perusal of these cases will satisfy the reader that they have not been selected on the ground of anything that indicated the approach of a spontaneously favorable change, or made them to differ from the ordinary character of cases of the classes to which they respectively belong." "The following conditions for making a selection from among those (cases) that should occur, for the purpose of publication, were considered advisable, namely, that they should not be of a slight nature, such as commonly yield with ease to confinement and restriction of diet,—that they should not include disorders previously subject to repeated spontaneous alternations of decline and increase,—and that there should be some reason to suppose that the persons subjected to the treatment were likely to give a fair trial in point of time and attention. Cases of pulmonary consumption, and most of those in which old organic disease was the apparent cause of the sufferings which existed, I did not think likely to furnish important results in general [and were therefore omitted.]" (Pref. pp. 53-55.)

As our object in the present article is not to expose the failures or blunbers of the homœopathists, but to endeavour to ascertain the truth, whatever it may be, respecting the alleged powers of homœopathy to cure diseases, we shall pass over all the cases in Dr. Henderson's list, which either tend to prove nothing, one way or other, or are more adverse than favorable to the claims of the new practice ; and we shall give our principal attention to those cases which the author himself must consider the best, inasmuch as many of them certainly seem, at first sight, to bear unequivocal evidence in favour of the treatment adopted.

The first twenty-four cases are examples of acute disease of slight severity, and supply no evidence, *pro* or *con*, worth quoting. They are principally cases of tonsillitis, dysentery, and erysipelas of the face. Every physician of experience would have expected them to get well under any treatment. It is but fair, however, to say, that they got well, as fast apparently under Dr. Henderson's treatment, as they would have done under ordinary medication. The twenty-fifth and twenty-sixth cases are well marked cases of acute rheumatism. They terminated in the short space of about five days, under the use of bryonia, assisted occasionally by aconite and belladonna, in doses of a billionth of a grain. The twenty-ninth and thirtieth cases are cases of severe neuralgia, the former speedily relieved after *one* dose of dulcamara, the latter, after *one* dose of aconite. It would be unfair to deny, that the result obtained in these four last cases would have been regarded as very satisfactory under any mode of allopathic treatment.

The thirty-third and thirty-fourth are cases of pneumonia. The first case proved fatal; but as the treatment was partly homœopathic partly allopathic, no inference can be drawn from it. The second was a well-marked case in a girl ten years of age. All the ordinary physical signs existed. Phosphorus and bryonia were the principal remedies administered, and the patient was convalescent about the ninth day.

After the large list of cases of pneumonia, successfully treated by Dr. Fleischmann, the result of this of Dr. Henderson's creates no surprise, and adds nothing to the strength of the evidence in favour of the homœopathic treatment of this disease. Such results may indeed astonish our heroic bleeders and mercurializers, or may even turn them, being so full of faith in drugs, to the pole opposite to heroism, homœopathy itself. But if mere recovery from an attack of pneumonia is to be admitted as evidence in favour of treatment, our heroes of the lancet and pill have other claimants for their suffrages besides the homœopathists. "In order to appreciate thoroughly (says M. Grisolle) the value of the various kinds of treatment cried up in pneumonia, it is indispensable that we should know accurately the progress, duration, and most frequent termination of it when treated purely on the expectant plan ; but we have not this medium of comparison. It is indeed true, that M. Biett treated during a whole year all the cases of pneumonia that came into his wards, with emollient drinks and cataplasms only, and the mortality was very inconsiderable. M. Magendie employs no other treatment in the same disease."* We may add that, to our knowledge, the same plan has been followed in one at least of the large hospitals of Germany, and the result was considered to have been far from unsatisfactory. And M. Grisolle informs us, that he himself, in the year 1840, treated eleven cases of pneumonia, "all perfectly characterized by the auscultatory phenomena, and by the expectoration," nearly in the same manner. The whole treatment consisted in confinement to bed, rigid diet, pectoral ptisans, and (rarely) a mild laxative such as castor oil. All the patients perfectly recovered, the mean term of convalescence being the 11th or 12th day. Dr. Henderson misjudges these cases in terming them "slight," in comparison with the one treated by him. They seem to have been fully as severe; and although M. Grisolle himself speaks of the symptoms as "sufficiently mild" to justify the experiment he undertook, they can hardly be regarded as slight, when we are told, that in nine out of the eleven cases, the disease "had reached the stage of red hepatization" before the treatment commenced.

Dr. Henderson's cases, No. 36 to 46 inclusive, are examples of headache, chiefly chronic, many severe, of long standing, and several of them rebellious to all former treatment. The results of the treatment of these cases are, upon the whole, very favorable; and several of the *cures* would, unquestionably, if occurring under ordinary treatment, have been regarded as not only striking but triumphant. We quote one of these.

"CASE XLIII.—*An Unmarried Lady, aged* 30.

"*1st April* 1845.—She is very spare and sallow, and subject to severe headaches. The pain affects the whole head, and is particularly intense on the right side and front, especially above the eyes. It is of a heavy compressive character, and accompanied by a sense of heat. Sometimes on the right side acute shootings occur. There is also much giddiness during the attacks.

"The sufferings come on in paroxysms, which last about twelve hours, and are particularly severe in the mornings, and attended with flushing often. Nausea and vomiting also accompany the attacks generally, and last for several hours. She is obliged to remain in bed while the paroxysms continue. Though they occur commonly in the morning, they are easily brought on by fatigue, and some-

* Traité Pratique de la Pneumonie, p. 560. † Ibid. p. 561.

times by even moderate exercise in the open air. She cannot endure a bright light or noise when they are present, without the sufferings being aggravated. *She is rarely more than two days free from the severe attacks, and has more or less headache every morning, which goes off after breakfast.* Bowels regular. She does not take medicine of any kind, as it never gives her relief. She sleeps well. The catamenia are regular, and occasionally excessive. Tongue clean. *She has been snbject to these attacks for above sixteen years, without any material difference during the whole time.* Bellad. 12, 2ce a day.

"9*th.*—She has had no severe headache since; indeed, any pain that has occurred has been so slight, that it is only on particular inquiry that she states she has had any. What has occurred has been very slight, only on one or two occasions, and for a very short period. No nausea or vomiting. Cont. Bellad.

"15*th.*—No severe attacks, and only slight headaches in the morning, ceasing after breakfast. Calcarea 6, 30, once a day.

"29*th*—The slight morning headaches continue. Sepia 30, once a-day.

"6*th May.*—The slight headaches have been very trivial, and for some days absent. No other ailment. Sepia 30, every second day.

"26*th.*—No headache of any kind since last report; a little confusion only on getting up. No nausea, &c. Omit Sepia

"2*d July.*—Has continued perfectly well." (pp. 121-2.)

Had this case of purely nervous headache come under the care of any of us allopathists, and had we prescribed for it "nervines," or "antispasmodics," or arsenic, steel, or other "tonics," according to our theories, our school, our experience, or our fancy, we should have certainly gained great credit from our patient and her friends for our "wonderful cure;" and some of us, doubtless, would have received it as our due. Had it taken place in the practice of a zealous and ambitious doctor under six-and-thirty, the probability is immense that it would have occupied a niche among the triumphs of medicine which crowd the weekly pages of our contemporaries. And we can see no sufficient reason why Dr. Henderson should not have his credit also. Philosophers, indeed, and hard-headed sceptics like ourselves, might demur to the claims of both; and might seek for an explanation of the facts beyond the limits of both *pathies.* Headaches and other nervous maladies do sometimes come to an end of their own accord; and as such an event is certainly *possible,* even immediately after the swallowing of a new drug, (and, possibly, *in consequence of the very swallowing,* and not the *drug,*) we leave it for the consideration and calculation of the wise, which of these two events was most probable: 1st, That the headache might have *chanced* to stop of its own accord on the very day it did, or was *charmed* away by the very *prestige* of homœopathy acting through the imagination; or, 2d, That one quadrillionth (1000000,000000,000000,000000,000000,000000*th*) of a grain of belladonna, and one decillionth (our printer has not *noughts* enough for this,) of a grain of the all-potent "sepia" did the feat.—*Non nobis est tantas.*

Cases 56 to 82 are examples of chronic disorders of the stomach and bowels, under the various forms of gastralgia, vomiting, dyspepsia, constipation, with headaches, &c. They are of the same general complexion, both as to character and result, as the cases of more formal headache. Several of them are certainly striking examples of rapid and most brilliant cures—cures, that is, just as good and as well authenticated as those of allopathy—*post hoc, ergo propter hoc.* We quote one case in illustration, chiefly because it is short.

"CASE LXXV.—*A Clergyman, aged about* 35.

"*6th May*, 1844.—Spare, and of ordinary complexion. Incapable of considerable exertion without fatigue. Had been subject to dyspepsia for a long time, and to irregularity of the bowels, which were habitually slow, and often constipated. *At length it became necessary for him to take a pill every second night, which he has continued to do habitually for above two years.* When so situated as to be unable to take his usual aperient, the bowels are confined for several days, and until he has recourse to medicine. Nux vom. 3, night and morning.

"*10th.*—Bowels began to act moderately on the 7th, and have been moved daily since, but not copiously. Cont.

"*19th.*—Bowels act daily without pills. Cont.

"*19th June.*—Has continued to take the Nux vom., and the bowels have been perfectly easy and regular.

"*21st August.*—Has taken no Nux vomica for six weeks, and the bowels have been in excellent order. His general health is better since commencing the treatment than for years before; his appearance is more robust and ruddy, and his strength is much improved.

"*January* 1845.—Has continued well in every respect." (pp. 171-2.)

Can anything in therapeutics surpass the evidence of the marvellous effect produced in this case by the 1000,000 part of a grain of nux vomica? One thing at least is certain, that the practice of Dr. Henderson conferred an inestimable benefit on his patient. But whether his *nux vom.* 3, was the cause or the occasion of this, is a question which may receive some elucidation from the contents of an admirable pamphlet published some years since by Dr. Henry of Dublin, entitled, 'A Dialogue between a Bilious Patient and a Physician,'* the object of which was to prove the evil of habitual medicine-taking, and the all-sufficiency of diet and exercise in such cases. Dr. Henderson's case may even have some light thrown on it from a much humbler source. Many years since, in the golden prime of our dispensary days, when we had remedies for every disease, and faith in many, we well remember, on one occasion, to have been not a little gratified and flattered by the brisk and cheerful response of a little girl to our morning query of "How do you do, my dear?" "Oh! I'm a great deal better *now*, Sir," said the little girl. Bravo! thought we, for our new mixture of seven ingredients—with its Basis, its Adjuvans, its Dirigens, its Corrigens, its Constituens! † "How long have you been so much better?" we asked, wishing to know the very hour of our triumph. "Since Friday, Sir," said the little girl. "Hah! what happened on Friday?" said we, musing as to the particular crisis which we had brought to pass. "Please Sir," said the little girl with a curtsey, "Please Sir, my medicine was done." And so, possibly, when this honest clergyman began to take the inconceivable *nux vom.* 3, the actual *medicine* which he had been swallowing for years *was*, like the little girl's, *done*.

Case 86 is a good one for the *post-hoc* school, whether homœopathic or allopathic :

"*A young lady aged* 19. August 5. For between two and three years has been subject to diarrhea with pain in the bowels, *after intervals rarely exceeding a week*. The attacks last for several days, and the bowels are moved from six to ten times a day. She is ill at present with one of them. [How many days has she been ill?] *Pulsat.* 6, *twice a day.* 29th. A day or two after last report the diarrhea ceased, and has not recurred. 10th Sept. Continues without having

* See this Journal, vol. VII, p. 476. † See Paris's Pharmacologia.

had a return of diarrhea; a length of interval which she *does not remember* to have occurred since the complaint began." (p. 181.)

When the intervals *did* exceed a week, how much did they exceed it? Did they ever reach four weeks? If the young lady could not *remember* this, Dr. Henderson should have made inquiry of those who could, before he adduced this flimsy case as evidence of the potency of his billionth of a grain of pulsatilla. Does Dr. Henderson think it a strange thing in the economy of nature, and only to be explained by the *Deus ex machina* of homœopathy, that a case of diarrhea, *characterized* by intervals of health, should stop *as usual,* although an incomprehensible something was given, and that it should not return for a few days longer on one particular occasion? These may seem little things to comment on, but surely little things will not be despised by the homœopathists of all men; and here they very significantly show the sort of philosophy we have to deal with. Men capable of admitting cases of this kind as evidence—and we could extract fifty from Dr. Henderson's book much feebler than this—are demonstrably disqualified to treat of things which demand for their handling the stern logic of a masculine mind.

While on the subject of diarrhea, we may here state a little fact not very irrelevant to the present discussion. Many years ago, when in charge of a large body of men in the public service, we had occasion to treat an epidemic diarrhea, of considerable violence but not dangerous. Finding our patients recover as fast under one as another of several methods of treatment adopted, we thought there would be no unpardonable *lèse-majesté* either to our royal master of London or our divine master of Delos, in carrying our trials one step further. Accordingly, we put half of our remaining patients on a course of orthodox physic, and half on homœopathic doses of flour (*farin.* 30) in the shape of bread-pills; and it puzzled us sadly to say which was the most successful treatment. *Query:* As there certainly *was* a decillionth of flour in each of our doses, and as this had undergone not a few "triturations and scrapings and shakings" in the barn, in the mill, on the crane, in the warehouse, in the joltings of a long land-journey, and in the infinitesimal vibrations of a ship in a long sea-voyage (we were then within the tropics), in the bakery, in the surgery, in the mortar (unglazed), on the slab, in the pill-box, in the patient's hand (with two arm-shakings,) in his mouth, in his throat, in his œsophagus,—who shall deny us the merit of having wrought our cures homœopathically? If it is asserted that *farina* is not found among the homœopathic remedies, we reply that charcoal is, and the *onus probandi* that the one is not as good as the other, lies with our opponents. If it is asserted that *farina* has not been "proved" on the healthy, and that it therefore comes not within the category of the *similia similibus,* we content ourselves with simply denying both assertions, and we pledge ourselves to produce, on trial, as many symptoms in a healthy man with the one as with the other. But even if our theoretical arguments should be rebutted, we take up our ground with Hahnemann and Dr. Henderson, and reply to all cavillers, that we have evidence beyond and above all theory—we have the irrefragable evidence of *facts.* For why? Have we not given our remedy, and has not a cure ensued? And "must not vain declamations be silent in the presence of infallible experience?"

It is unnecessary to proceed with the examination of Dr. Henderson's cases. They are all of the same general character; and the minutest analysis of them would not alter the conclusion to which the portion already commented on, infallibly lead. This conclusion is similar to that we drew from the cases of Dr. Fleischmann. In the present case we shall state it in the words of Dr. Henderson himself:—

"I can hardly conceive," says Dr. Henderson, "that those who are better entitled to judge, will find it difficult to admit, on the supposition that the cases have been exactly as related, that there has been a proportion of success among them with which they would have been fully satisfied, as the result of the ordinary means." (p. 49.)

Whether we may be ranked among those "who are better able to judge," or not, we do not know; but we do not hesitate to declare, that the amount of success obtained by Dr. Henderson in the treatment of his cases, would have been considered by ourselves as very satisfactory, had we been treating the same cases according to the rules of ordinary medicine.

In making these admissions in respect to the instances of treatment supplied by Drs. Fleischmann and Henderson, we wish formally to guard ourselves against being supposed to admit, at the same time, as if it were one and the same thing, or as if the one was a corollary of the other, that the result of the homœopathic treatment *generally*, is, and will be, as successful as the result of the ordinary treatment generally. It is *possible* that this may be the case; but, as we have no certain evidence that it is so, it would be absurd on our part to assume that this is the fact. We wish to keep strictly within the record, which goes no further than this, that a certain definite number of cases of disease, treated homœopathically by these two gentlemen, appear to have had as successful results as if they had been treated allopathically, or according to one or other of the prevailing modes of ordinary practice. No documents are in existence calculated to lead to a judgment of the *general question* at issue between the two doctrines.

But many of our readers, we expect, will be of opinion that, in admitting what we have done, we are betraying the cause of legitimate medicine, and lending our aid to extend the heresy of homœopathy. If such should be the result of our admissions, we cannot help it. We have said only what we believe to be true; and if what we believe is in reality the truth, the promulgation of it cannot lead to evil. Truth is good. If the art of medicine, as we profess and practise it, cannot bear investigation, and shrinks before the light of truth, from whatsoever quarter it may come, it is high time that it should cease to be sanctioned and upheld by philosophers and honest men. If, on the contrary, it be true and good—even if it be only but partially true and moderately good—the stirring touch of inquiry and the stimulus of opposition cannot fail to benefit it in the end.

What, then, it will naturally be asked, is the explanation of the momentous fact we have announced, that a considerable number of diseases have been, and perhaps continue to be, treated as successfully by homœopathists as by allopathists? *Is it, that the one kind of treatment is as good as the other?* IS IT, THAT HOMŒOPATHY IS TRUE?

To both of these queries we give an unequivocal and decided negative, so far, at least, as this can be given in a case where we have, as yet, no demonstrative proof on one of the sides of the question. We may, indeed, have proof sufficient to satisfy any reasonable mind, that the theory or doctrines, or principles of homœopathy are false; but, as yet, we have no demonstrative evidence that it is false in its practical bearings—false, that is, powerless, as a means of curing diseases. It will not be disputed by any one conversant with the history of medicine, that these two things are not only distinct, but independent of each other. We can, however, assert with the greatest positiveness, that, as far as the evidence supplied by the documents now before us, or the evidence we have been able to gather from other published writings of the new school, goes,—there exists not a tittle of actual PROOF that homœopathy is true in this aspect. On the other hand, we have not a little positive evidence to prove that it has often failed to cure in cases where, according to its principles and the alleged experience of its professors, it ought to have cured, and in which allopathy did effect a cure. Still, this is only negative proof, and might be accounted for or explained away on grounds that would not necessarily compromise the existence of homœopathy as a means of cure. In a case so extraordinary, so marvellous, it may be said, as that of homœopathy, nothing short of the most positive and demonstrative evidence of its curative powers can be accepted; nothing, in short, will suffice but the *experimentum crucis* of a comparative trial, on the large scale, of *its* powers, on the one hand, and of *nature's* powers, on the other. Until it can be proved by clinical experience on an extended field, and on two parallel groups of similar diseases, that homœopathy cures better than nature, we are warranted by every principle of philosophy, not merely in doubting, but in denying its truth.

It would be easy to give numerous and strong reasons for the necessity of insisting on this extreme degree of evidence in the case of homœopathy. We shall only here advert to a single one. If, for the sake of argument, we were to admit that homœopathy were partially true, and, therefore, that it might fairly be received as one of the recognized methods of treating diseases, it would appear to us, according to our present light, to be very unfortunate for medicine if this were done. The guiding principles of homœopathy appear to us to be of that character, which must render its exercise very injurious to medicine as a branch of science. Based, as it is, on mere extrinsic, secondary phenomena, or symptoms, and exclusively engaged in the search for and adaptation of specific remedies to such phenomena, we cannot but regard it as calculated to destroy all scientific progress in medicine, and to degrade the minds of those who practise it. Its direct tendency seems to be that of severing medicine from the sciences, and establishing it as a mere art, and thus converting physicians from philosophers to artisans. Of course, if, by such a conversion, diseases were to be better treated and more speedily and frequently cured, it would be not only absurd, but transcendently wicked so to sacrifice the welfare of humanity for the sake of a scientific phantom; but, as we have said, it is anything but proved that such a result would follow the change, and therefore, until the proof is obtained, it behoves all who regard the prosperity and dignity of true art, to resist its progress.

But, such being our estimate of the character and powers of homœopathy, on what principle can we explain the fact, above admitted, that diseases have been cured and continue to be cured, alike under its ministration as under that of ordinary practice? *Is it, that* ALLOPATHY *is false also? Or is it, that, to obtain an explanation of the fact, we must pass by both, and fix on some* THIRD POWER, *coincident with both, yet belonging to neither?*

We cannot give to these queries, as to the former, either a simple negative, or simple positive reply. In answer to the first, we would say, that allopathy is certainly *true*, in a limited sense, that is to say, it unquestionably possesses, to a certain extent, the power of curing diseases. It is, however, *not true*, in an absolute sense, or in the sense in which it is regarded by some, inasmuch as it does *not* cure a great proportion of the diseases it is supposed to cure. In answer to the second, we admit that there is a third power, common to or coincident with both, which, while it explains all the triumphs of homœopathy, reduces those of allopathy within much narrower limits than its more zealous votaries are wont to assign it: *this is* THE POWER OF NATURE.

And here we must be permitted to enter into a little detail; as the placing this subject in its true light appears to us a matter of great importance, not merely in relation to the main object of the present discussion, but in its bearings on the subject of Practical Medicine generally, and especially on the momentous question of its improvement, or, if we may be allowed to say so, its REFORMATION, which we think is impending.

Much confusion and difficulty have been thrown over our consideration of the question of the nature and powers of homœopathy, and many disturbing and distorting influences here come into play in our attempts to form a just estimate of the value of allopathy, because of our misappreciation, on the one hand, of the actual powers of nature in freeing the body from the diseases that arise in it, and because of misappreciation, on the other hand, of the powers of art in working to the same end.

Health is such a blessing and disease such an evil, that the existence of the desire to get rid of the latter, and thus to recover the former, must be coextensive with the possession of reason by the organism that suffers. Strongly to desire is equivalent to the origination of action to gratify the feeling. Hence the origin of the medical art, which must have been coeval with the origin of man himself; hence the conception and formation of plans for the purpose of relieving pain, and of theories to account for and explain them, springing up in the mind of the first sufferers, and growing in number and variety from that time to the present; hence the constant interference of art with the natural processes of disease in the human body. When in process of time, medicine came to be established as a distinct profession, such interference necessarily became much more frequent and much greater; until, at length, the result was, that *all* diseases occurring in civilized communities, were interfered with as a matter of course. In the long succession of human generations, almost everything possible, physical or moral, was at one time or other tried, with the view of proving its possession or non-possession of remedial powers. The necessary consequence has been, the fixing in the minds of men, not merely of the professors of the medical art but of mankind in general, these two notions, first,—that nature was inadequate to the cure of most diseases, certainly of severe diseases; and, secondly, that art was adequate. And these notions have not only come down to us as heirlooms of physic, but have been almost universally received as axioms, without investigation, both by the medical profession and the public. The result of all this has been, that the members of the medical profession at all times, and more especially in modern times, have been kept in a state of forced ignorance of the natural progress and event of diseases; in other words, of the true *natural history of diseases* in the human body; and they have been and continue to be almost as ignorant of the actual power of remedies in modifying, controlling, or removing diseases, and from the self-same cause, viz., that as art has almost always been permitted to interfere in the morbid process, it has been impossible to say what part, if any, of the result was attributable to nature, or what part to the remedies employed.

And yet, that nature *can* cure diseases without assistance from art, is a fact demonstrated by evidence of the most unequivocal kind, and of almost boundless extent. It suffices here to refer cursorily to a few of the more open sources of such evidence.

1. The cure of diseases among uncivilized nations of ancient and modern times, under the sole influence of magic, charms, or other practices equally ineffective.

2. The general treatment of diseases in the ruder and simpler times of physic, as recorded in the writings of the early fathers of our art.

3. The record of innumerable cases in the works of medical authors, more particularly before the eighteenth century, in which, from various causes, no medical treatment, or one demonstrably powerless, was employed.

4. The records of the *Expectant system* of medicine, long and extensively prevalent in various parts of Europe; also of other analogous systems of practice in vogue at different times in various countries, which could exert no substantial influence on disease or on the animal economy.

5. The wide-spread and frequently the exclusive employment, more especially in modern times, of universal, or as they are now called, quack medicines, under the use of which almost all curable diseases have frequently got well. Whether these medicines consist of inert substances, or of substances of positive medicinal power, the inference derived from their employment is nearly the same. All of them have, most indubitably, *cured* (to use this word in its common acceptation) a vast number of diseases; and whether the event was consequent on the use of a substance of no real power, or possessing *a particular power only*, must be allowed to be nearly the same thing. In our own day we have seen many large fortunes made in this country by the sale of various patent drugs of this kind—from Solomon's Balm of Gilead to Parr's Life Pills; and this fact alone proves their *real efficacy*, that is, proves it on the very same grounds of evidence admitted in legitimate medicine. Success, that is, the apparent cure of diseases on an extensive scale, could alone keep up a sale of them so extensive as to enable their proprietors to accumulate large fortunes. And of this kind of success—that is, the getting-well of patients under their use, according to the legitimate *post-hoc* mode of reasoning, every medical man must have witnessed many instances.

6. The now fashionable system of Hydropathy furnishes strong and extensive evidence of a like kind, although on somewhat different grounds. This mode of treating diseases is unquestionably far from inert, and most opposed to the cure of diseases by the undisturbed processes of nature. It, in fact, perhaps affords the very best evidence we possess of the curative powers of art, and is, unquestionably, when rationally regulated, a most effective mode of treatment in many diseases. Still it puts, in a striking light, if not exactly the curative powers of nature, at least the possibility, nay facility, with which all the ordinary instruments of medical cure (drugs) may be dispensed with. If so many and such various diseases get well entirely without drugs, under one special mode of treatment, is it not more than probable, that a treatment consisting almost exclusively of drugs, may be often of non-effect, sometimes of injurious effect?

An intelligent and well-educated hydropathical physician, on whose testimony I can entirely rely, informs me, that in a great many cases that have come under his care in a hydropathic establishment, he has observed the symptoms amend on the first commencement of hydropathic remedies, with a suddenness and speed which he could not conscientiously ascribe to the influence of the means used, but which rather appeared to result from the abandonment of injurious drugs which the patients had previously been in the habit of taking. In some cases, to test this point, the physician purposely abstained from treating the patients at all, and yet witnessed the same marked amendment. My informant points out to me another natural field of observation in this line, in the numerous patients discharged, cured, or relieved, from hydropathic establishments, almost all of whom carry with them such a horror of drugs that they never have recourse to them, if it can be helped, afterwards. Yet these people recover from their subsequent diseases—even without Hydropathy!

7. Mesmerism, also, we think, must come either within the category of cases illustrating the curative powers of nature, or, at least, the non-necessity of drugs, or both.

8. We may next instance a large and important class of cases, in which some philosophical physicians, in all times, have instituted direct experiments, both publicly and privately, to test the powers of nature, by either withholding all means of treatment, or by prescribing substances totally inert; the result often being the cure of many diseases under such management.

9. Lastly, we must advert to what is, perhaps, the most extensive and valuable source of all—the actual practice of the more scientific physicians of all ages, in the latter part of their career,—men of philosophic minds as well as of much experience. It is well known, from the history of physic, that a large proportion of men of this class have, in their old age, abandoned much of the energetic and perturbing medication of their early practice, and trusted greatly to the remedial powers of nature. The saying of a highly respected and very learned physician of Edinburgh, still living at an advanced age, very happily illustrates this point. On some one boasting before him of the marvellous cures wrought by the small doses of the Homœopathists, he said, "this was no peculiar cause for boasting, as he himself had, for the last two years, been curing his patients with even less, viz., with nothing at all!"

The candid consideration of what precedes will, we hope, go far to satisfy the minds of most men, of the justness of the conclusion previously come to by us—viz. That the curative powers of nature suffice to explain all the triumphs of homœopathy: we think the consideration of some additional influences essentially connected with the exercise of the new system, must entirely remove all doubt on the subject. We will here specify a few of these influences.

1. The abandonment of all previous medication, often, doubtless, of injurious influence on the malady; and the free field thus left for the operation of the *vis medicatrix*:

> "For Nature then has room to work her way;
> And doing nothing often has prevailed,
> When ten physicians have prescribed and failed."

2. A much stricter regulation of the diet and regimen, including the entire omission of vinous and other alcoholic drinks, nervous and other stimulants, as tea, coffee, pepper, &c.

3. The influence of imagination, stimulated by previous belief of the potency of the remedies, and nourished by fervent faith, hope, &c. Of course we know that this argument will not apply to the cases of infants, and that very small number of patients who are not made aware of the nature of the treatment to which they are subjected. As an equivalent, at least, to the latter part of this objection, we can adduce the fact, repeatedly established, of the non-effect of the homœopathic remedies, when experimentally administered by allopathic practitioners, without the knowledge of the patient.

4. The indirect influence of this faith, hope, &c. in inducing patience, so that time is allowed for nature to work the cure in her own way. And here we would remark, that the establishment of this most desirable state of mind—itself, by the way, directly curative—is an event much more likely to occur in homœopathic than in allopathic practice. The conscientious homœopathist, who believes he has selected the proper remedy, must possess a degree of confidence in the result of his medication, which an ordinary practitioner can be but rarely justified in feeling. Acting on the principle of specifics, the former can wait in patience for the event, which is that of the subsidence of the disease simply; while the latter, acting, for the most part, only indirectly on the disease, and obliged to vary his means according to circumstances, is sure, in every long disease, more or less, to lose patience and entertain doubt; and the betrayal of such feelings to the patient (and they can hardly be concealed) has a most unfavorable effect on the cure, by destroying faith, confidence, and hope. It may be added, that these most desirable states of mind are much more likely to arise and be retained where, as in homœopathic practice, *no obvious effect* on the system is seen, or expected to be produced by the medicines, than where, as in ordinary practice, a decided and visible effect is produced, and yet no amendment ensues.

But while we are thus exalting the powers of nature at the expense of homœopathy, are we not, at the same time, laying bare the nakedness of our own cherished allopathy? If it is nature that cures in homœopathy, and if homoeopathy (as we have admitted) does thus cure, in certain cases, as well as allopathy, do we not, by this admission, inevitably expose ourselves defenceless to the shock of the tremendous inference,—that the treatment of many diseases on the ordinary plan must, at the very best, be useless; while it inflicts on our patients some serious evils that homœopathy is free from, such as, the swallowing of disagreeable and expensive drugs, and the frequently painful and almost always unpleasant effects produced by them during their operation? This inference, and the dilemma it involves, are always held up by the homœopathists *in terrorem* to any allopathist who should think of using the argument of nature's *autocrateia* against their system; and they think the threat too terrible to be encountered with disregard, much less with defiance, by any man in the actual practice of allopathy.

"If the latter be true [the negative quality of homœopathic practice] what a fearful judgment," say Drs. Drysdale and Russel, "must necessarily be formed of the allopathic method! . . . This view is one of momentous consequence to the practitioners of medicine; and we trust it will have its due effect in leading them seriously to reflect on their responsibility—on the awful circumstances in which they voluntarily place themselves." (p. 235.)

"It is often said," adds Dr. Henderson, "that the benefits of homœopathy flow mainly from the omission of medicine altogether, of which the system is supposed by its opponents in reality to consist. This opinion had better be reconsidered, if it lead to the practical inference, as I think it does, that some 80 or 90 per cent. of the patients who employ medical practitioners would be better off without them." (p. 237.)

These threats do not deter us from accepting the horn of the dilemma presented to us; nor do we think it worth while cavilling about the precise amount of the estimate involved in Dr. Henderson's inference. This may or may not be accurate; we believe that it is exaggerated; but be this as it may, we concede at once to him the truth of his general proposition; *and still adhere to* ALLOPATHY. In doing so, we consider that though we are embracing a system extremely imperfect, we are, at least, embracing one which, with all its faults, contains a considerable amount of truth, and a yet greater amount of good; and which, above all, is, or may be made, in its exercise, consonant with the principles of science, and is capable of indefinite improvement; while, in rejecting homœopathy, we consider that we are discarding what is, at once, false and bad—useless to the sufferer and degrading to the physician.

So much for HOMŒOPATHY—for the present at least. It only now remains for us to add a few momentous words on ALLOPATHY—words which have long had their prototypes in our thoughts, but which now find formal utterance for the first time, forced from us, as it were, by the immediate pressure of the important discussion in which we have been engaged. Before entering, however, on this concluding portion of our task, we must be allowed to make an explanatory remark, relating partly to something that precedes as well as to what is to follow. The reader will now understand the precise meaning of an expression we made use of in the commencement of this article, to the effect,—that Homœopathy would probably be the cause of more important fundamental changes in the practice of medicine, than any previous system since the days of Galen. In repeating our belief of the correctness of this statement, we will add, that in this respect, if in no other, the doctrine of Hahnemann will have conferred an inestimable benefit on the healing art. Regarding Homœopathy, as it will now be seen we do, as a system of medicine, which essentially leaves diseases to the operations of nature, we must consider it as having been the means of instituting a grand natural experiment in therapeutics, which, though of vital importance to our art, could not have been compassed by any other means we know of. From the results of this experiment, conducted as it is on the most extensive scale, and likely to be prolonged through an indefinite period, we have the prospect of obtaining at last, a true natural history of human diseases, and the means of ascertaining the actual powers of nature in relieving or removing them; and, as a corollary of this knowledge, the real powers of art in the same field. We may also hope to learn from the same source, directly or indirectly, the proper occasions for applying and withholding the instruments by which art works, and to discriminate accurately the effects produced by one class of operations from those produced by the other; so as to come, at length, to something like an appreciation of the true powers and actions of remedies, of which, at present, we are lamentably ignorant. In truth, a portion, though only a small portion, of this most important knowledge has been already obtained from the experiment; just enough to show us more clearly than before, the extent of our ignorance,—the first step to knowledge. We hope this will appear from the brief exposition of the present state of Therapeutics which we are now to make.

In finishing our examination of the writings of the Homœopathists, we said, that we did not shrink from admitting and adopting the inferences—however unfavorable to Allopathy — which seemed necessarily to flow from the results of their treatment of diseases. The principal of these inferences have been already stated more than once. It seems necessary, however, to recapitulate the more important of them here. These are:—

1. That in a large proportion of the cases treated by allopathic physicians, the disease is cured by nature, and not by them.

2. That in a lesser, but still not a small proportion, the disease is cured by nature, in spite of them; in other words, their interference opposing, instead of assisting the cure.

3. That, consequently, in a considerable proportion of diseases, it would fare as well, or better, with patients, in the actual condition of the medical art, as more generally practised, if all remedies, at least all active remedies, especially drugs, were abandoned.

We repeat our readiness to admit these inferences as just, and to abide by the consequences of their adoption. We believe they are true. We grieve sincerely to believe them to be so; but so believing, their rejection is no longer in our power; we must receive them as facts, until they are proved not to be so.

Although Homœopathy has brought more signally into the common daylight this lamentable condition of medicine regarded as a practical art, it was one well known before to all philosophical and experienced physicians.

It is, in truth, a fact of such magnitude,—one so palpably evident, that it was impossible for any careful reader of the history of medicine, or any long observer of the processes of disease, not to be aware of it. What, indeed, is the history of medicine but a history of perpetual changes in the opinions and practice of its professors, respecting the very same subjects—the nature and treatment of diseases? And, amid all these changes, often extreme and directly opposed to one another, do we not find these very diseases, the subject of them, remaining (with some exceptions) still the same in their progress and general event? Sometimes, no doubt, we observe changes in the character and event, obviously depending on the change in the treatment,—and, alas, as often for the worse as for the better; but it holds good as a general rule, that, amid all the changes of the treatment, the proportion of cures and of deaths has remained nearly the same, or, at least, if it has varied, the variation has borne no fixed relation to the difference of treatment.

In making this statement, we are far from denying that practical medicine has made considerable progress since it was first established as an art, or that we do not now cure more diseases and save more lives than our forefathers did. The truth of our assertion,—taken as a general assertion, and when the question is regarded in the only way it ought to be regarded, in an approximative, not in an absolute sense,—is not thereby in any respect invalidated. We do not deny that medicine has made progress, or that it can cure diseases and save life;—we merely assert that the *superiority in the proportion* of the instances in which it does so, in the present day, is most lamentably small, all things considered, when placed side by side with the amount of any former day. In several of our commonest and most important diseases, it is hardly to be questioned that the proportion is little, if at all, on our side, and in others it is manifestly against us.

This comparative powerlessness and positive uncertainty of medicine, is also exhibited in a striking light, when we come to trace the history and fortunes of particular remedies and modes of treatment, and observe the notions of practitioners, at different times, respecting their positive or relative value. What difference of opinion, what an array of alleged facts directly at variance with each other, what contradictions, what opposite results of a like experience, what ups and downs, what glorification and degradation of the same remedy, what confidence now—what despair anon in encountering the same disease with the very same weapons, what horror and intolerance at one time of the very opinions and practices which, previously and subsequently, are cherished and admired!

> "Quod fuit in pretio, fit nullo denique honore;
> Porro aliud succedit, et e contemptibus exit,
> Inque dies magis appetitur, floretque repertum
> Laudibus, et miro 'st mortaleis inter honore."

To be satisfied on this point we need only refer to the history of any one or two of our principal diseases or principal remedies, as, for instance, fever, pneumonia, syphilis; antimony, bloodletting, mercury. Each of these remedies has been, at different times, regarded as almost specific in the cure of the first two diseases; while, at other times, they have been rejected as useless or injurious. What seemed once so unquestionably, so demonstrably true, as that venesection was indispensable for the cure of pneumonia? And what is the conclusion now deducible from the facts already noticed in the present article, (p. 246,) and from the clinical researches of Louis and others?* Is it not that patients recover as well, or nearly as well, without it?—Could it have been believed possible by the practitioners of a century since, that syphilis could be safely treated and successfully cured without mercury? or that it could ever be questioned that mercury was not specific in the cure of this disease? And yet what are the opinions and the practices of the surgeons of the present day, and the indubitable facts brought to light during the last thirty years? Are they not, that mercury is not necessary (speaking generally) to the cure of any case, and that it is often most injurious, in place of being beneficial? The medical god, Mercury, however, seems as unwilling to be baulked of his dues, as the mythological; if he has lost the domain of syphilis, he has gained that of inflammation; and many of our best practitioners might possibly be startled and shocked at the supposition, that their successors should renounce allegiance to him in the latter domain, as they themselves had done in the former. And yet such a result is more than probable, seeing that there exists not a shadow of more positive proof (if so much) of the efficacy of the medicine in the latter than in the former case.

The same truth, as to the uncertainty of practical medicine generally, and the utter insufficiency of the ordinary evidence to establish the efficacy of many of our remedies, as was stated above, has been almost always attained to by philosophical physicians of experience in the course of long practice, and has resulted, in general, in a mild, tentative, or expectant mode of practice in their old age, whatever may have been the vigorous or heroic doings of their youth. Who among us, in fact, of any considerable experience, and who has thought somewhat as well as prescribed, but is ready to admit that,—in a large proportion of the cases he treats, whether his practice, in individual instances, be directed by precept and example, by theory, by observation, by experience, by habit, by accident, or by whatsoever principle of action,—he has no positive proof, or rather no proof whatever, often indeed very little probability, that the remedies administered by him exert any beneficial influence over the disease? We often may hope, and frequently believe, and sometimes feel confident, that we do good, even in this class of cases; but the honest, philosophical thinker, the experienced, scientific observer, will hesitate, even in the best cases, ere he commit himself by the positive assertion, that the good done has been done by him. When physicians of this stamp have met in consultation in any doubtful case, and when they have chanced to be startled out of their conventionalities by the bold doubt, or bolder query, of some frank brother of the craft, has not the confession, like the confidence, been mutual?

> "And when his comrade's thought each *doctor* knew,
> 'Twas but his own, suppress'd till now, he found."

From these our free confessions and bold denunciations of the feebleness and uncertainty of therapeutics, it may possibly be inferred, that we are entirely sceptical of the truth of medicine as a science, and think most

* See Louis' Recherches sur les Effets de la Saignée, Paris 1835; or the Review of the work in this Journal, Vol. I, p. 97.

meanly of it as a practical art. And yet this is not so. On the contrary, we look upon medicine, regarded in all its parts and all its bearings, as a noble and glorious profession, even in its present most imperfect state; and we believe it destined to become as truly grand and glorious in actual performance, as it now is in its essence, its aims, and its aspirations.

It is an unquestionable truth that medicine, both as a science and as an art, is, on the whole, progressive; and its progress, compared with that of preceding times, has been immense during the last sixty years. In the fundamental parts of the science, in physiology, pathology, and diagnosis, great and manifold additions have been made to our knowledge, during this period; several positive improvements have been also introduced into the general mode of treating our patients; and we have acquired one or two unequivocal accessions to our stock of certain means of relieving or curing diseases. We believe we may also add, with truth, that the general style of practice in this country has become better, is less guided by theory and tradition, is more discriminating, less confident and bold, less perturbing and meddling. We have learned a good deal at home, and still more, perhaps, from home. The long peace and the general intercourse of nations consequent thereon, have permitted every country to know what every other country possesses. British medicine has thus profited considerably, and most especially by the importation of some of the humbler notions and milder practices of our continental neighbours. In the treatment of acute diseases, we have attained somewhat nearer to the heroic virtue of patience, from an increased knowledge of the morbid processes going on; in chronic disorders we have become more regimenal and less druggish; in all cases, perhaps, we have grown a little more trustful of nature, and a little less trustful of art. Nevertheless, it cannot be denied that the more ordinary proceedings of a large proportion of the practitioners in this country differ from those of their predecessors, much more in their nature than in their effects; and that they are, to a lamentable extent, palpably and egregiously wrong. We doubt, therefore, if we should greatly, if at all, exceed the bounds of truth, if we said, that the progress of Therapeutics, during all the centuries that have elapsed since the days of Hippocrates, has been less than that achieved in the elementary sciences of medicine, during the last fifty years. This department of medicine must, indeed, be regarded as yet in its merest infancy. It would, doubtless, be going far beyond the truth to assert, that there is no certainty in medicinal therapeutics, and that the whole practice of medicine, in as far as this consists in the administration of drugs, is a system of traditionary routine and conventionalism, hap-hazard, and guess-work; but it is not going beyond the truth to assert, that *much* of it is so. In the hands of men of scientific education, men of philosophical views and long experience, and who, from the position they occupy and the confidence they inspire, are enabled to proceed exactly as they think best, Practical Medicine, we readily admit, is, even now, a rational and wise system, rarely productive of evil if it fails to benefit, and often benefiting in the highest degree. But in the hands of those who are differently circumstanced in every respect; who either travel contentedly in the broad highways of tradition, or deviate into still more dangerous paths which they deem rational; who, confounding therapeutics with medicinal formulæ, prescribe according to rule or according to fancy,—medicine is a very different art, and its practice productive of very different results.

The foregoing elucidations, it will not be doubted, disclose a lamentable state of things; but it is not a state to be despaired of; much less is it one to be concealed as something disgraceful. It is more our misfortune than our fault that it is as it is; but if it were our fault, still it ought to be made known. Here, as in morals, the more sensibly we feel our defects, the more openly and heartily we confess them, the more likely are we to get rid of them. As thus reflected in our critical mirror, the features of our Ancient Mother assuredly look somewhat unattractive. She seems neither happy nor prosperous; yea, she seems sick, very sick; yet not sick unto death. On the contrary, we believe that she is more vivacious and vigorous than at any preceding time; her countenance is merely "sicklied o'er by the pale cast of thought," from the strength of her inward throes; "the genius and the mortal instruments are now in council, and her state, like to a little kingdom, is suffering the nature of an insurrection." And such, in truth do we believe to be, literally, the condition of physic at this moment. Things have arrived at such a pitch, that they cannot be worse. They must mend or end. We believe they will mend. The springs of life are yet untouched; the constitution retains its rallying power; the vis medicatrix is in action; and we flatter ourselves that there is yet enough of young blood and energy and wisdom in our ranks, to redeem the past, and to achieve that glorious REGENERATION, which has been long announced by infallible signs and portents in these later days. Old as we are, we yet hope to see raised the standard of "YOUNG PHYSIC," though we cannot expect to see it furled, after the destined victory is won.

The course of our subject would now lead us to attempt to show wherein the defects and evils of the present system of practical medicine mainly consist; the causes of these; and the means that seem best calculated to remove them;—with a view to the substituting a better system in its place. But this must be reserved for another occasion, if not for another hand. Our space, for the present is overpast; our time expended; and the reader's patience, we suspect, already sufficiently tried. Had we proceeded in our task, it was our design to show, that the changes in therapeutics contemplated by us had nothing to do with any dogma, or system, or crotchet of our own; but were merely such alterations, or if the word be allowed, such reforms, in the existing state of things, as seemed necessarily to flow from a consideration of the more obvious deficiencies heretofore exposed. Such as it is, our project of reform, is, at the least, as conservative as radical. We are not altogether discontented with the general principles on which medicine, considered as a science, is now studied by many men of education; we do not object greatly to the mode in which we know it to be exercised as an art by many of its professors; but we cannot shut our eyes to the enormous mass of its defects, or to the grievous evils which result, both to the community and the profession, from the way in which it is carried out in detail, by a large proportion of existing practitioners. It is with a view to the removal of these defects and evils—as far as they are removable—that we would fain excite the interest and claim the attention of our readers, and, through them, of the profession generally.

It would be presumptuous in us, in the present stage of the question, to attempt to give even a formal Outline or Sketch of the Reform in Practical Therapeutics which appears so necessary, and which we believe to be impending. This is a work which can only be the result of mature reflection, and of the labour of many years and many hands. All which we can think of attempting at present, is to set down, almost at random, a few of the various considerations that press upon us, touching the many things to be thought of and done, the manifold evils to be abated, the manifold benefits to be achieved, by the enthusiastic and active spirits whom we have heretofore sportively personified under the name of "YOUNG PHYSIC," and to whom we look with confidence for the consummation of the great REFORMATION which assuredly will come.

In submitting these suggestions to criticism, we would request, that their extemporaneous and undigested character be borne in mind. All of them, we believe, to be true and just; many of them of high importance; and although more mature reflection may prove some of them, at least, to be neither the one nor the other, we shall, nevertheless, not regret having written them. They may excite others to consider this momentous subject, and thus elicit from better minds, thoughts worthier of remembrance and fruitful of greater things.

### COGITANDA—EXCOGITANDA—AGENDA.

1. To endeavour to ascertain, much more precisely than has been done hitherto, the natural course and event of diseases, when uninterrupted by artificial interference; in other words, to attempt to establish a true Natural History of human diseases.

2. To reconsider and study afresh the physiological and curative effects of all our therapeutic agents, with a view to obtain more positive results than we now possess.

3. To endeavour to establish, as far as is practicable, what diseases are curable and what are not; what are capable of receiving benefit from medical treatment and what are not; what treatment is the best, the safest, the most agreeable; when it is proper to administer medicine, and when to refrain from administering it; &c. &c.

4. To endeavour to introduce a more philosophical and accurate view of the relations of remedies to the animal economy and to diseases, so as to dissociate in the minds of practitioners the notions of *post hoc* and *propter hoc.*

The general adoption by practitioners in recording their experience, of the system known by the name of the *Numerical Method,* is essential to the attainment of the ends proposed in the preceding paragraphs, as well as in many that are to follow.

5. To endeavour to banish from the treatment of acute and dangerous diseases, at least, the ancient axiom, *melius anceps remedium quam nullum,* and to substitute in its place the safer and wiser dogma—that where we are not certain of an indication, we should give nature the best chance of doing the work herself, by leaving her operations undisturbed by those of art.

6. To endeavour to substitute for the monstrous system of Polypharmacy now universally prevalent, one that is, at least, vastly more simple, more intelligible, more agreeable, and, it may be hoped, one more rational, more scientific, more certain, and more beneficial.

7. To direct redoubled attention to hygiene, public and private, with the view of preventing diseases on the large scale, and individually in our sphere of practice. Here the surest and most glorious triumphs of medical science are achieving and to be achieved.

8. To inculcate generally a milder and less energetic mode of practice, both in acute and chronic diseases; to encourage the Expectant preferably to the Heroic system,—at least where the indications of treatment are not manifest.

9. To discountenance all active and powerful medication in the acute exanthemata and fevers of specific type, as small pox, measles, scarlatina, typhus, &c., until we obtain some evidence that the course of these diseases can be beneficially modified by remedies.

10. To discountenance, as much as possible, and eschew the habitual use (without any sufficient reason) of certain powerful medicines in large doses, in a multitude of different diseases, a practice now generally prevalent and fraught with the most baneful consequences.

This is one of the besetting sins of English practice, and originates partly in false theory, and partly in the desire to see manifest and strong effects resulting from the action of medicines. Mercury, iodine, colchicum, antimony, also purgatives in general and bloodletting, are frightfully misused in this manner.

11. To encourage the administration of simple, feeble, or altogether powerless, non-perturbing medicines, in all cases in which drugs are prescribed *pro forma,* for the satisfaction of the patient's mind, and not with the view of producing any direct remedial effect.

One would hardly think such a caution necessary, were it not that every-day observation proves it to be so. The system of giving and also of *taking* drugs capable of producing some obvious effect,—on the sensations, at least, if not on the functions,—has become so inveterate in this country, that even our *placebos* have, in the hands of our modern doctors, lost their original quality of harmlessness, and often please their very patients more by being made unpleasant!

12. To make every effort not merely to destroy the prevalent system of giving a vast quantity and variety of unnecessary and useless drugs, (to say the least of them,) but to encourage extreme simplicity in the prescription of medicines that seem to be requisite.

Our system is here greatly and radically wrong. Our officinal formulæ are already most absurdly and mischievously complex, and our fashion is to double and redouble the existing complexities. This system is a most serious impediment in the way of ascertaining the precise and peculiar powers (if any) of the individual drugs, and thus interferes, in the most important manner, with the progress of therapeutics.

We are aware of the arguments that are adduced in defence of medicinal combinations. We do not deny that some of these combinations are beneficial, and therefore proper; but there cannot be a question as to the enormous evils, speaking generally, resulting from them. Nothing has a greater tendency to dissociate practical medicine from science, and to stamp it as *a trade*, than this system of pharmaceutical artifice. It takes some years of the student's life to learn the very things which are to block up his path to future knowledge. A very elegant prescriber is seldom a good physician. And no wonder. Tailors, barbers, and dancing-masters, however learned they may be in the externals of gentility, are not expected to be fine gentlemen or men of fashion.

13. To endeavour to break through the routine habit, universally prevalent, of prescribing certain determinate remedies for certain determinate diseases or symptoms of diseases, merely because the prescriber has been taught to do so, and on no better grounds than conventional tradition.

Even when the medicines so prescribed are innocuous, the routine proceeding impedes real knowledge by satisfying the mind, and thus producing inaction. When the drugs are potent, the crime of mischief-making is superadded to the folly of empiricism. In illustration, we need merely notice the usual reference, in this country, of almost all chronic diseases accompanied with derangement of the intestinal functions, to "affection of the liver," and the consequent prescription of *mercury* in some of its forms. We do not hesitate to say, that this theory is as far wrong as the practice founded on it is injurious; we can hardly further enhance the amount of its divarication from the truth.

14. To place in a more prominent point of view the great value and importance of what may be termed the physiological, hygienic, or natural system of curing diseases, especially chronic diseases, in contradistinction to the pharmaceutical or empirical drug-plan generally prevalent. This system, founded as it is on a more comprehensive inquiry into *all* the remote and exciting causes of disease, and on a more thorough appreciation of *all* the discoverable disorders existing in all the organs and functions of the body, does not, of course, exclude the use of drugs, but regards them (generally speaking) as subservient to hygienic, regimenal, and external means,—such as the rigid regulation of the diet, the temperature and purity of the air, clothing, the mental and bodily exercise, &c., baths, friction, change of air, travelling, change of occupation, &c. &c.

15. To endeavour to introduce a more comprehensive and philosophical system of Nosology, at least in chronic diseases, whereby the practitioner may be led less to consider the name of a disease, or some one symptom or some one local affection in a disease, than the disease itself,—that is, *the whole* of the derangements existing in the body, and which it is his object to remove, if possible.

16. To teach teachers to teach the rising generation of medical men, that it is infinitely more *practical* to be master of the elements of medical science, and to know diseases thoroughly, than to know by rote a farrago of receipts, or to be aware that certain doctors, of old or of recent times, have said that certain medicines are good for certain diseases.

17. Also to teach students that no systematic or theoretical classification of diseases, or of therapeutic agents ever yet promulgated, is true, or anything like the truth, and that none can be adopted as a safe guide in practice. It is, however, well that these systems should be known; as most of them involve some pathological truths, and have left some practical good behind them.

18. To endeavour to enlighten the public as to the actual powers of medicines, with a view to reconciling them to simpler and milder plans of treatment. To teach them the great importance of having their diseases treated in their earliest stages, in order to obtain a speedy and efficient cure; and, by some modification in the relations between the patient and practitioner, to encourage and facilitate this early application for relief.

19. To endeavour to abolish the system of medical practitioners being paid by the amount of medicine sent in to their patients; and even the practice of keeping and preparing medicines in their own houses.

Were a proper system introduced for securing a good education to chemists and druggists, and for examining and licensing them—all of easy adoption—there could be no necessity for continuing even the latter practice; while the former is one so degrading to the medical character, and so frightfully injurious to medicine in a thousand ways, that it ought to be abolished forthwith, utterly and for ever.

20. Lastly, and above all, to bring up the medical mind to the standard necessary for studying, comprehending, appreciating, and exercising the most complex and difficult of the arts that are based on a scientific foundation,—the art of Practical Medicine. And this can only be done by elevating, in a tenfold degree, the preliminary and fundamental education of the Medical Practitioner.

Such are a few of the labours in store for our young Hercules of Physic; a few samples of the varied contents of the stable he is called upon to cleanse; and a few pailfuls, it may be, of the veritable Alpheian he is to work withal:

"———— Mox in ovilia
Demisit hostem vividus impetus;
Nunc in reluctantes dracones
Egit amor————————."

---

